# Paternal microbiota manipulation influences offspring microbial colonization and development in a sex role-reversed pipefish

**DOI:** 10.1038/s41598-025-16222-y

**Published:** 2025-08-22

**Authors:** Kim-Sara Wagner, Frédéric Salasc, Silke-Mareike Marten, Olivia Roth

**Affiliations:** 1 Marine Evolutionary Biology, Zoological Institute, Kiel University, 24118 Kiel, Germany; 2 OSU Institut Pytheas, Aix-Marseille Université, Marseille, 13009 France

**Keywords:** Evolution, Microbiology

## Abstract

Microbes are acquired through vertical and environmental horizontal transmission. Vertical transmission is directly linked to reproductive success and entails early transmission, facilitating coexistence of host and microbes over generations. The multiple potentially interacting routes of vertical transmission are challenging to be disentangled in conventional sex-role species, as they are mostly intermingled on the maternal side, i.e., through egg production, pregnancy, birth and postnatal care. The evolution of male pregnancy in syngnathids (pipefishes and seahorses) offers an opportunity to separate vertical microbial provisioning through the egg (maternal) from provisioning through pregnancy (paternal). We experimentally evaluated the existence and role of paternal vertical microbiota provisioning through male pregnancy on offspring development and microbial colonization. Male pipefish were exposed to antibiotics, and subsequently recolonized with bacteria of paternal, maternal, and environmental origin (spike treatment). After pregnancy, the microbiota of developing offspring was characterized using 16 S rRNA sequencing of the V3-V4 region. Paternal antibiotic and spike treatments influenced the microbial composition of the brood pouch and offspring microbiome development. Paternal spike treatment shortened pregnancy duration and enhanced offspring survival, underlining its beneficial effect for early life stages. Expanding on how distinct vertical microbial transmission routes shape the offspring microbiome will foster our understanding of holobiont function in health and disease.

**Supplementary Information:**

The online version contains supplementary material available at 10.1038/s41598-025-16222-y.

## Introduction

**Fig. 1:**
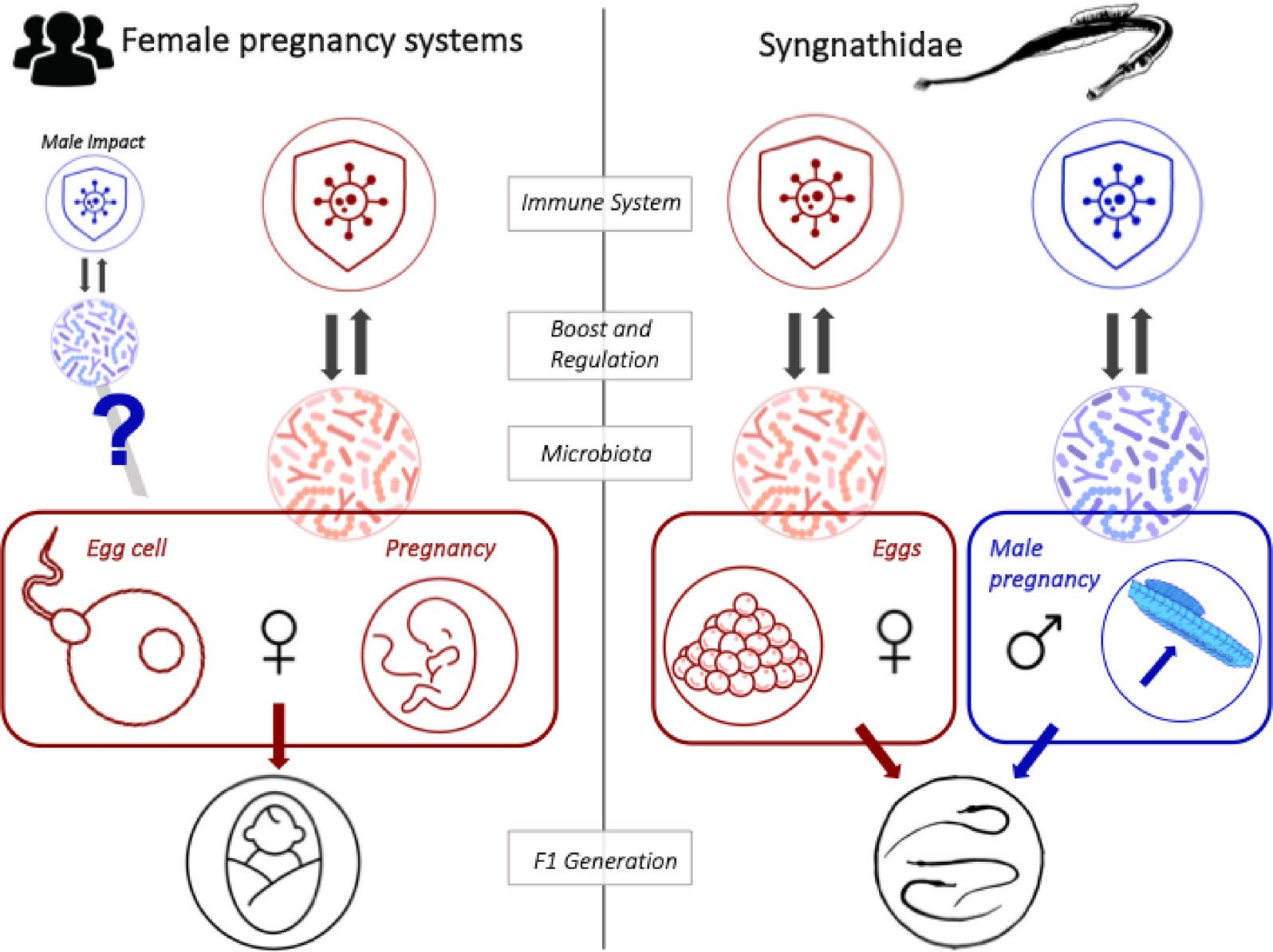
Immune system and microbiota transfer across generations: Comparison of classic female pregnancy systems (left) with unique male pregnancy in the pipefish *Syngnathus typhle* (right) with a focus on the interaction of the microbiota and the immune system.

The interplay of the host with its microbiome can be adaptive; facilitating long-lasting interactions that shape host development, digestion, immune regulation, behaviour, and pathogen protection^1–3^. To understand the interactions of the host with its microbiome and their functional consequences on host development, we need to unravel the microbial composition starting from the initiation of their colonization. Vertical transmission fosters the initial microbial colonization as early as during egg development^4^and can continue through the transfer in intimate parent-offspring interactions, such as pregnancy and postnatal parental care^5–7^. Vertical transmission routes are manifold and mostly bound to the maternal side impeding the disentangling of their roles in offspring microbial colonization and development (Fig. 1, left). Therefore, the intertwining of egg production and pregnancy within the female body confounds the investigation of vertical transfer^8^leaving us behind with open questions about the significance of the specific microbes transferred during egg production, pregnancy or postnatal parental care.

Throughout vertebrate evolution, viviparity — the development of the embryo inside the parental body — has evolved independently in over 150 lineages^9^. While in almost all cases the female is the pregnant sex, the syngnathids, a group of teleost fishes, represent the unique evolution of male pregnancy^10^. In the broad-nosed pipefish, *Syngnathus typhle*, the female deposits her eggs into the semi-enclosed brood pouch of the male where they are fertilized, protected, and nourished until the male gives birth^11^. This characteristic reproductive strategy provides the opportunity to disentangle the typically intermingled vertical microbial transmission through eggs and pregnancy: we expected mothers to transfer microbes *in ovo*, while fathers were expected to transfer microbiota to offspring during pregnancy through the brood pouch (Fig. 1, right)^8^. In contrast, horizontal transfer was suggested to occur only when the brood pouch becomes strained and permeable during late pregnancy^8,12^ allowing to independently study the timing and role of vertical microbial transfer in depth.

The microbiome of *Syngnathus typhle* differs in composition between the female gonads and the male brood pouch^12^. While the maternal microbiota was suggested to shape the offspring gut microbiome, paternal microbes are expected to rather influence the establishment of the offspring whole-body microbiome^8^. We aimed to provide the first experimental assessment of paternal microbial transmission through male pregnancy, by manipulating the paternal brood-pouch microbiome and disseminating its impacts on offspring microbiome and health. We depleted the paternal natural brood-pouch microbiome with antibiotics, and subsequently recolonized the brood pouch with selected natural bacterial isolates before mating males with untreated females. We hypothesized that this paternal microbial manipulation would influence offspring microbiome establishment and offspring development. Given the intricate interplay of father and offspring during pregnancy, we expected paternal-specific microbes to successfully colonize the male tissue and to be transferred to the offspring. This study enhances the functional understanding of host-microbiota interactions in animals with intense parental care, such as pregnancy, and provides insights into the transmission and establishment of microbiota across generations.

## Methods

To ultimately assess the transfer of microbes through paternal pregnancy, we conducted three experiments: **(1)** Cultivation and characterization of sex-specific microbiota, **(2)** effectiveness of antibiotics for depleting the natural pipefish brood-pouch microbiome, and **(3)** manipulating paternal sex-specific microbes to unravel their impact on the offspring. In all three experiments, we sequenced the 16 S rRNA gene^13^ with Sanger sequencing (experiment 1) and with Illumina MiSeq Amplicon (V3/V4 region) (experiment 2 & 3). Given our focus on potential changes in microbial composition rather than absolute bacterial loads, we analyzed relative abundances, as MiSeq 16 S rRNA amplicon sequencing does not permit direct quantification of microbial communities. This approach, however, was chosen to compare shifts in community structure and assess differences in microbial composition across experimental conditions. Data processing, visualization, and statistical testing were performed in R Studio^14^.

### Ethics declarations

The work was carried out in accordance with the relevant guidelines and regulations provided by the German animal welfare act (TierSchG) and with the ethical approval given by the *Ministerium für Landwirtschaft*,* ländliche Räume*,* Europa und Verbraucherschutz des Landes Schleswig-Holstein* (MLLEV-SH, *Ministry of Agriculture*,* Rural Areas*,* Europe and Consumer Protection*) (permit no. 63 − 7/18). No wild endangered species were used in this investigation and all methods are reported in accordance with ARRIVE guidelines.

## Fish catching and rearing protocols

Broad-nosed pipefish (*Syngnathus typhle*) were caught in the bay of Orth (Fehmarn, Germany) (54°26’ N, 11°02’E). At the Helmholtz Centre for Ocean Research Kiel (GEOMAR), they were separated by sex, treated with formalin (1:8000 solution) for one hour on three consecutive days to remove potential ectoparasites, and kept in Baltic Sea water flow-through systems until allocation into the experimental facilities (16:8 day-night; 15 PSU).

## (1) Cultivation and characterization of sex-specific microbiota

We aimed to cultivate the sex-specific microbiota of *Syngnathus typhle* from female eggs, male brood pouches throughout pregnancy and environmental samples. The findings were compared to a previous cultivation-independent NGS study^12^. In total, 65 fish were used for the cultivation study: 20 females, 17 non-pregnant, 16 early pregnant and 12 late pregnant males. Animals were euthanized with tricaine methane sulfonate (MS-222, 500 mg l^−1^; Sigma-Aldrich, St. Louis, USA) followed by weight and length measurements. Microbiota samples were isolated from six groups: female gonads (FG), non-pregnant males: placenta-like tissue of the developing brood pouch (NP), early pregnant males: placenta-like tissue (EPP) and whole juveniles (EPJ) as well as late pregnant males: placenta-like tissue (LPP) and whole juveniles (LPJ). Tissue samples were homogenized in autoclaved phosphate buffered saline (PBS) and plated onto Marine Broth (MB, Carl Roth GmbH, Karlsruhe, Germany) or *Vibrio*-selective TCBS agar plates (Carl Roth GmbH). After incubation, bacterial colonies were prepared for amplification of the 16 S rRNA (primers 27 F and 1492R). The PCR products were purified and Sanger sequenced^15^. Raw sequences were aligned and edited with CodonCode Aligner (V8.0.2). Consensus sequences were exported and submitted to NCBI’s BLAST for taxonomic identification within the 16 S ribosomal RNA sequences (Megablast algorithm)^16^. The identified bacteria were compared to the sex-specific indicator species from the cultivation-independent study^12^. Differences between stages and tissues were analyzed using PERMANOVA and ANOSIM on a ranked dissimilarity matrix and calculated with the Jaccard similarity coefficient (999 permutations). A multiple correspondence analysis (MCA), followed by pairwise Adonis analyzed microbial clusters of sexes, respectively pregnancy stages.

## (2) Effectiveness of antibiotics for natural pipefish microbiota depletion

### Microbiota sample collection

We assessed the effectivity of antibiotics in depleting the natural sex-specific microbiota. Following a randomized, fully reciprocal study design, we used 64 *Syngnathus typhle* (32 males, 32 females) and exposed them to antibiotics for 312 h (13 days), consisting of 48 h treatment, followed by 274 h maintenance (reduction of antibiotic concentration by 75%). Pipefish were treated with chloramphenicol (treatment: 40 mg/L, maintenance: 10 mg/L), kanamycin (treatment: 10 mg/L, maintenance: 2.5 mg/L), a mixture of both antibiotics with identical concentrations^17,18^ or they were left untreated (control). Every antibiotic treatment was replicated in 8 tanks containing one male and one female each. Gut and sex-specific microbiota were sampled at 0, 6, 12, 24, 48, 192 and 312 h. Gut microbiomes were sampled by gastric swabs using a sterile paper point (ANTÆOS^®^ Absorbent Paper Points sterile, size 15/20/25, VDW GmbH), following a method described in a study about the pipefish gut microbiome^19^. For assessing the reproductive sex-specific microbiomes, the inside of the brood pouch (male) and the surface of the ovipositor (female) were swabbed with sterile cotton swabs (Copan Italia, Thermo Fisher Scientific). Tank water antibiotic concentrations were assessed with agar well diffusion tests on *Epibacterium mobile* lawns (prevalent in pipefish, susceptible to both antibiotics applied)^20^. Both the antibiotic treatment and the gastric swabbing did not induce any incidences of death. From the total of 64 experimental fish (32 males, 32 females), half were sampled after 48 h, and the remaining half after 312 h. At each of the two time points four pipefish per treatment and sex were euthanized (MS-222) and sampled for final gastric as well as reproductive sex-specific microbiota swabs. DNA was extracted using DNeasy 96 Blood & Tissue Kit (QIAGEN GmbH, Hilden, Germany) with enzymatic lysis buffer pre-treatment. The 16 S rRNA V3/V4 was sequenced over Illumina Miseq amplicon sequencing at the Institute of Clinical Molecular Biology (IKMB, Kiel, Germany).

### Data analysis and statistics

From demultiplexed paired-end fastq files, sequences were merged, quality filtered, and analyzed using QIIME2 version 2019.10^21^. Chimeras were removed using the “consensus” method within DADA2^22^. Sequences were truncated 50 bases from forward and 70 bases from reverse reads in both runs. For taxa comparison, relative abundances based on all obtained reads were used. The Naïve Bayes classifier was trained on the SILVA138 99% OTUs full-length sequence database^23^ and sequences from our dataset were assigned to ASVs from SILVA database with similarity on the 99% level. The phyloseq package v.1.46.0^24^ was used for further analyses and data visualization. The data set was subdivided for the reproductive sex-specific (brood pouch and ovipositor) and gut microbiomes, with a particular focus on the male brood pouch microbiota as it serves as our target tissue in the microbiota manipulation conducted in the consecutive experiment 3. Count data was normalized, log-transformed and prevalence filtered to only preserve ASVs that are present in > 20% of the samples, thus visualizations display log relative counts. Alpha diversity indices were estimated for Shannon. Indicator species analysis using MULTIPATT with 999 permutations was performed using the R package indicspecies^25^ to identify ASVs that were significantly associated with specific treatment groups. This method assesses the association strength of each taxon with one or more groups based on their relative abundance (proportion within a sample) and frequency of occurrence (how consistently it appears across samples). PCAs and PCA biplots were calculated and visualized with FactoMineR^26^. A mixed ANOVA (factors: treatment x time) tested for differences in Shannon diversity between treatment groups over time points. A repeated measured PERMANOVA evaluated the impact of treatments on sex-specific microbiota composition (beta diversity) over time and a MANOVA on ordination axes was performed to test for treatment differences at each time point separately.

## (3) Manipulating paternal sex-specific microbiota to unravel impact on the offspring

**Fig. 2:**
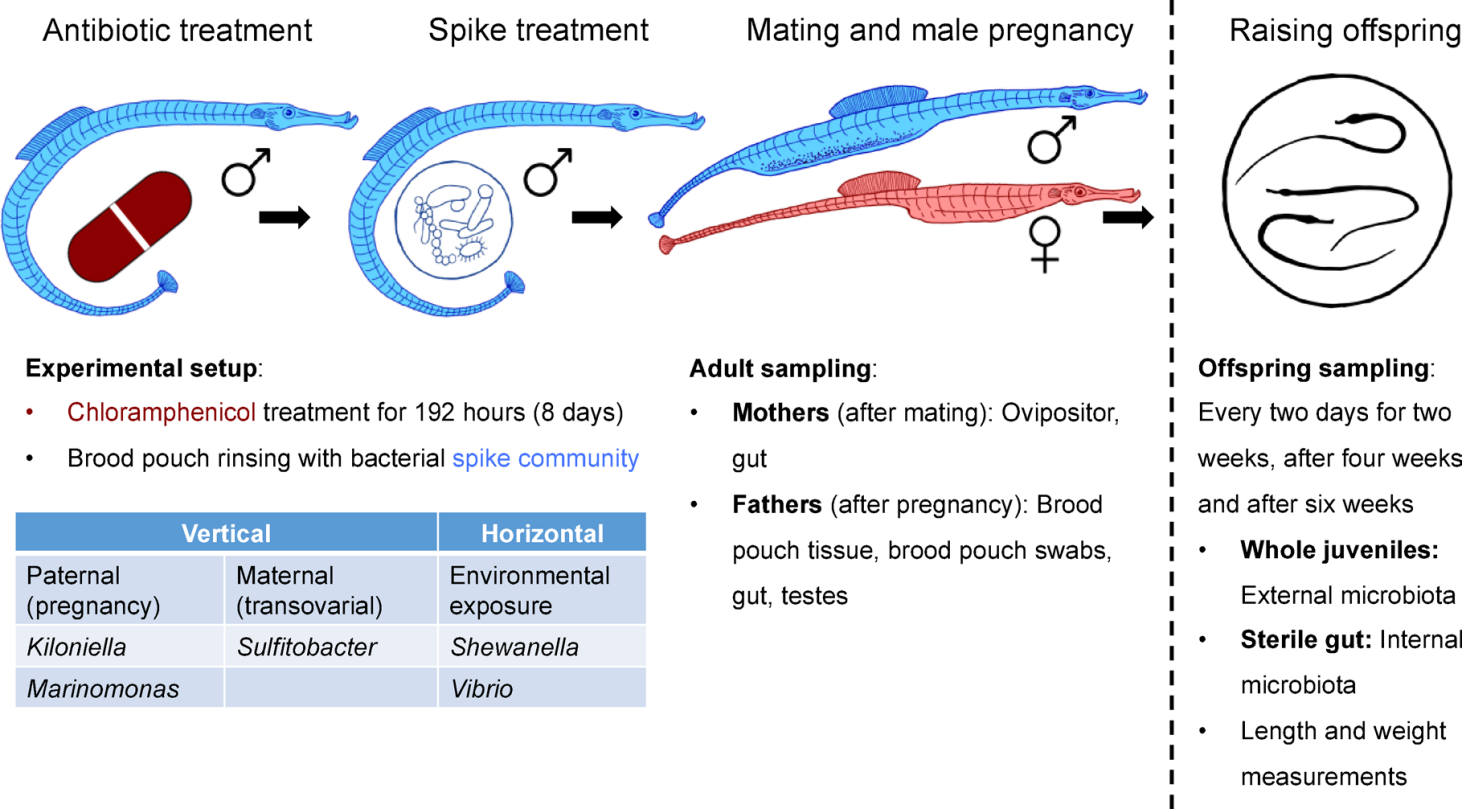
Setup of experiment 3: Manipulating paternal brood pouch microbiota to assess the impact on offspring development and early microbial colonization. Fathers received a treatment (antibiotics and/or spike) and were mated with untreated females. The bacterial strains used in the spike represent different transmission routes: *Kiloniella*, *Sulfitobacter*, and *Marinomonas* were selected as indicators of vertical transfer via maternal or paternal tissues, while *Shewanella* and *Vibrio* represent typical horizontally acquired, environmentally associated strains. Offspring were kept in separate tanks and were repeatedly sampled for 45 days.

In the last experiment, the methods established in the two previous experiments were applied: randomly selected male pipefish were fully reciprocally treated with antibiotics (chloramphenicol) and recolonized with candidate strains for vertical and horizontal transfer (spike treatment) (Fig. 2). We aimed to assess the potentially interacting impact of paternal antibiotic and spike treatment, on microbial colonization and offspring development.

## Antibiotic treatment of male fish

72 *S. typhle* (36 males, 36 females) were used in this experiment. 18 *S. typhle* males were allocated to the antibiotic treatment group and exposed to chloramphenicol (48 h 40 mg/L, followed by 144 h 10 mg/L), while the other 18 male individuals were left without antibiotic treatment (details: experiment 2). Females and untreated males were kept in separate circulation systems. Water temperatures were slowly increased from 11 °C to 17 °C. All fish were fed live *Mysid* spp.; however, for the antibiotic-treated males, the mysids were immersed in chloramphenicol (40 mg/L) for 1 h prior to feeding to ensure consistency in antibiotic exposure.

### Spike treatment of male pipefish, pregnancy, and microbiota sampling of adults

Prior to mating, half of the male pipefish received a spike-bacteria treatment resulting in four different treatment groups: Control, Antibiotics (antibiotics applied, no spike bacteria added), Spike (no antibiotics applied, spike bacteria added) and AntiSpike (antibiotics and spike treatment). The spike community consisted of five selected bacterial strains isolated from *S. typhle* or their environment (seawater) and cultivated during experiment 1: *Sulfitobacter* (vertical maternal transfer candidates), *Kiloniella* and *Marinomonas* (vertical paternal transfer candidates), *Shewanella* (horizontal transfer candidate) and *Vibrio* (prevalent in *S. typhle* and its environment) (Table 1). Overnight cultures were separately diluted to OD600 0.5, washed with phosphate-buffered saline (PBS), mixed equally and 30x concentrated (Supplementary Fig. 8). Brood pouches of half of the males were flushed with 50 µl spike culture. The other half was flushed with 50 µl PBS (sham control). After treatment, pipefish pairs were placed in 80 L glass tanks, each divided by a filter mat foam separator to house two pairs. After mating, females were removed and samples taken for gut and ovipositor microbiome sequencing. After two weeks of pregnancy, the male fish were moved into individual 20 L tanks. Immediately after birth, placenta-like tissue, brood pouch surface, hind gut, and testes were sampled from the male pipefish. Tissues were flash frozen and stored at −80 °C.

**Table 1:** Spike bacteria strains and information about the tissue they were isolated from. Right column indicates whether the strain was selected as an indicator for paternal, maternal or environmental transfer.

Spike	Tissue	Sex	Fish ID	Indicator
*Kiloniella*	Brood pouch	Male	ST1	Paternal
*Marinomonas*	Brood pouch	Male	ST52	Paternal
*Sulfitobacter*	Eggs	Female	ST12	Maternal
*Shewanella*	Sea water		*MB/TCBS agar*	Environment
*Vibrio*	Sea water		*MB/TCBS agar*	Environment

### Juvenile time series

10 juveniles from each batch were moved to 400 ml beakers for daily monitoring of survival for 45 days (Fig. 2). From the remaining offspring (kept in their birth tanks), length and weight were recorded and microbiota was sampled directly after birth, and then every two days for two weeks, after four weeks (28 days) and six weeks (45 days). Tissues were stored at −80 °C. The fish were fed daily with live artemia. At each sampling, two offspring per tank were sacrificed (one for whole-body and one for gut microbial 16 S sequencing). In addition, on each sampling day, seawater of each tank (*n* = 237) was collected and the fish food (*n* = 15) was sampled using sterile paper points. DNA was extracted and sequenced from all tissue and water samples for 16 S rRNA sequencing as in experiment 2. We here only discuss relevant time points (complete dataset: supplementary spreadsheets 6 and 7).

### Data analysis

Paternal treatment effects on gestation length and number of offspring were assessed with a one-way ANOVA. To assess the effect of paternal treatment on juvenile performance, we analyzed survival, weight, and size development separately. Survival was evaluated using a Cox proportional hazards model with treatment, age, and their interaction as predictors to account for time-dependent variation in mortality. In addition, we applied an ANCOVA to test for treatment and age effects on survival rates at given time points. Estimated marginal means with pairwise contrasts (Tukey-adjusted) were used to explore differences between treatment groups. For weight and length, we conducted separate ANCOVAs including treatment, age, and their interaction, followed by post hoc pairwise comparisons using estimated marginal means. Processing of raw 16 S rRNA amplicon sequences using the QIIME2 workflow followed the procedures described above. Truncation lengths were: run 63: forward 250, reverse 195; run 64: forward 260, reverse 190; run 65: forward 275, reverse 220; run 79: forward 250, reverse 195. Whole microbiome composition was assessed with a PCA biplot and MANOVA, followed by ANOVA. To identify sequences stemming from the recolonization spike culture, QIIME2 sequences were aligned to the sequences of the spike community. Relative abundance of spike community strains within the microbiome were analysed with a generalized least square model (GLS) using normalized, log-transformed counts. Differences between treatment groups were assessed with a Kruskal-Wallis test followed by a Dunn’s post-hoc test.

## Results

### Comparing cultivable and sequenced sex-specific microbiota for experimental manipulation

We aimed to compare the isolated, cultivated and characterized sex-specific microbiota 16 S rRNA Sanger sequences of *Syngnathus typhle* to the NGS dataset from a cultivation-independent study^12^facilitating the selection of strains suitable for manipulating the paternal sex-specific microbiota (experiment 3). 767 full length 16 S rRNA sequences were trimmed (clip ends) and assembled, resulting in 376 consensus sequences (presence/absence matrix, species and genus level). From 79 tissue samples (13 female gonads (FG), 16 non-pregnant (NP), 11 early pregnancy pouch (EPP), 10 early pregnancy juveniles (EPJ), 12 late pregnancy pouch (LPP), 12 late pregnancy juveniles (LPJ)), 92 strains from 38 genera were identified. This corresponds to approximately 2.97% of the total number of 3090 OTUs (97% cut-off) identified in the NGS approach^12^. Another six bacterial isolates from four genera were isolated from the seawater. Microbiome composition differed between stages (ANOSIM (*p* = 0.011); PERMANOVA (*p* = 0.001)): FG – LPJ: *p* = 0.020; NP – LPJ: *p* = 0.015; EPP – LPJ: *p* = 0.015; EPJ – LPJ: *p* = 0.023 (pairwise Adonis, Supplementary Table 2).

The MCA visualized associations between sexes or developmental stages with the 20 mainly contributing bacterial isolates (Supplementary Fig. 1). Dimension 1 drove the microbiota of the female gonads (FG) away from the remaining five developmental stages, whereas dimension 2 segregated the microbiota associated to the late pregnancy stage (LPP, LPJ) from the other stages. Microbiota associated with non-pregnant (NP) males and the early pregnancy stage (EPP, EPJ) showed the strongest overlap. This pattern resembles the pattern identified by the previous NGS study^12^. *Vibrio* predominantly distinguished female eggs from other tissues, while *Pseudoalteromonas* was overrepresented in male brood pouches, particularly towards late pregnancy, as well as in juveniles (Supplementary Fig. 1). In the NGS study, female eggs were represented by *Brevinema* and *Halomonas*, whereas the male brood pouch was characterized by *Marinomonas*, *Kiloniella*, *Ruegeria* and *Pseudoalteromonas*^12^ (Complete dataset: supplementary spreadsheet 1).

For manipulating the sex-specific microbiota of *S. typhle* through antibiotic treatments followed by recolonization, we pooled five bacterial strains with a potential role in transgenerational transfer and early microbial colonization (hereafter called the “spike community”). These strains were isolated from *S. typhle* tissues (experiment 1) and selected based on their presence in both culture-dependent and culture-independent analyses, ensuring that they were not only representative of the natural pipefish microbiota but also cultivable for experimental manipulation. While the composition of sex-specific microbiota was broadly consistent between sequencing and cultivation approaches, not all key taxa identified as indicator species in the NGS study^12^ were successfully cultivated. Therefore, we focused on strains that were represented within the 2.97% cultivable fraction of the sex-specific (female gonads and male brood pouch) microbiota, allowing for direct experimental validation of their functional role. For paternal vertical transfer, we included *Kiloniella* and *Marinomonas*, which were enriched in the male brood pouch microbiome and exhibited dynamic changes across pregnancy stages. For maternal transfer, we incorporated *Sulfitobacter*, a strain identified as indicator associated with female gonads and the brood pouch microbiota during late pregnancy, suggesting a potential maternal contribution to the offspring microbiome^12^. *Shewanella* and *Vibrio*, bacterial taxa commonly found in aquatic environments, were included to account for environmentally derived microbial exposure.

Although our experimental approach was necessarily limited to cultivable strains and the spike community represents only a fraction of the natural microbiota, this targeted selection enabled a first step toward investigating the relative influence of maternal, paternal, and environmental microbial sources during early colonization when applied exclusively paternally.

### Antibiotic effects on overall microbial diversity and on the selection of strains for paternal transfer (spike community)

**Fig. 3:**
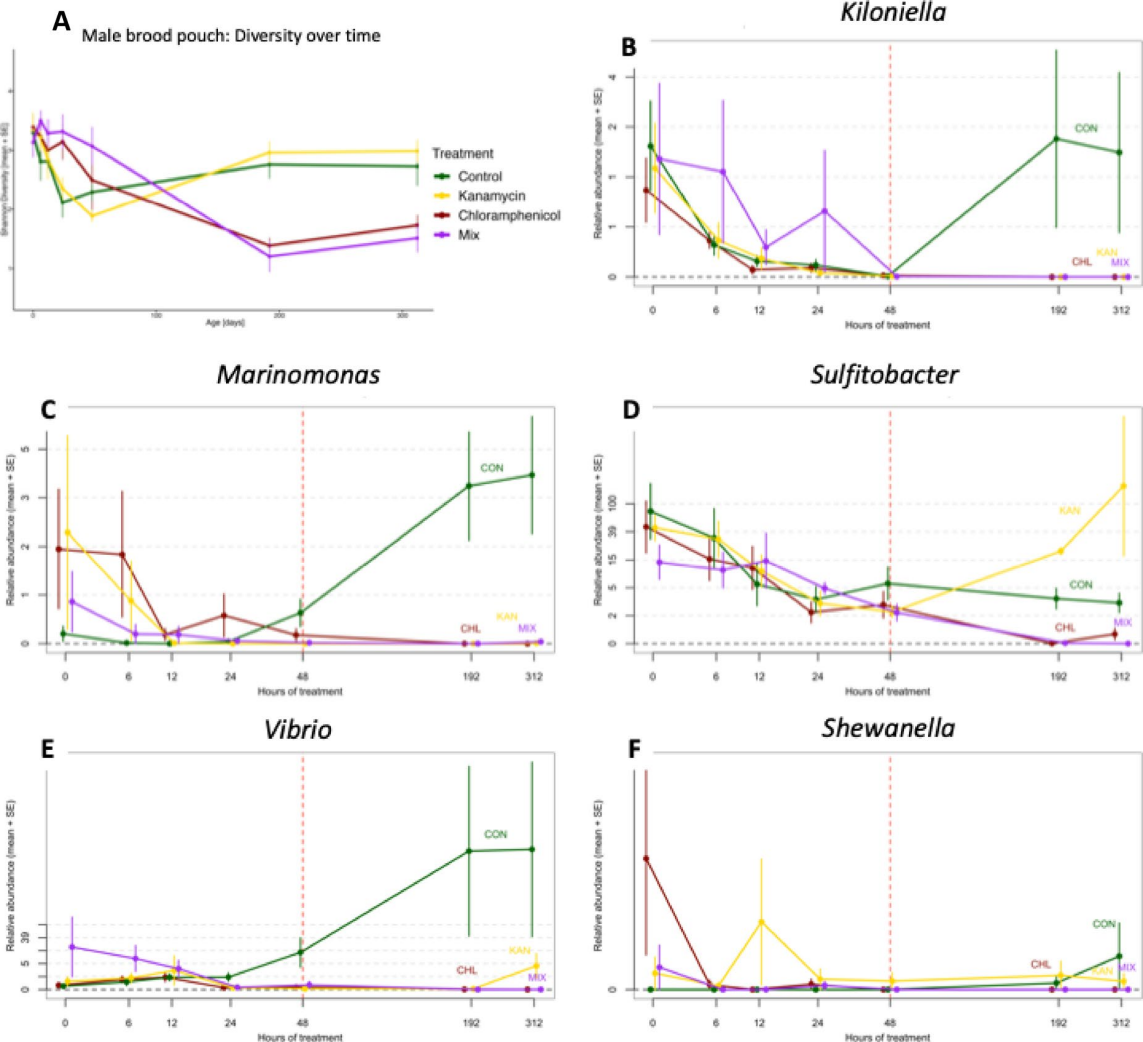
Impact of antibiotics (mean + SE) on Shannon diversity of male brood pouch microbiota (**A**) and on relative abundance of spike community strains within the brood pouch tissue (**B**-**F**) over a period of 312 h. Colours indicate treatment groups: Control (green), Kanamycin (yellow), Chloramphenicol (red) and Mix (purple). Red vertical line represents time of treatment dose (48 h after treatment start). Samples were taken at the time points indicated on the x-axis; data points are horizontally shifted to display individual variation and avoid overlap.

This experiment aimed to investigate how antibiotic treatments influenced sex-specific microbial diversity of *S. typhle* (males: brood pouch, females: ovipositor surface). 754 samples were submitted to 16 S rRNA MiSeq amplicon sequencing. A total of 19,837,424 reads were returned (mean run 47: 28,517 reads/sample, mean run 48: 21,022 reads/sample), along with 10,688 representative sequences (Supplementary Table 1). The duration of the antibiotic treatment (time) as well as time x antibiotic interaction influenced the male brood pouch microbiota alpha diversity (Shannon) (ANOVA Type III: treatment: *p* = 0.894; time: *p* < 0.001; time x treatment: *p* < 0.001). No significant difference in alpha diversity were identified for timepoints T0 (p.adj = 1), T6 (p.adj = 1), T12 (p.adj = 1) and T48 (p.adj = 0.656) whereas from T24 (p.adj = 0.04) onwards, diversity indices decreased (T192: p.adj = 0.001; T312: p.adj = 0.024). Within the first 48 h (treatment), alpha diversity fluctuated (Fig. 3A). After 8 days (maintenance), diversity was lowest in the treatments Chloramphenicol and Mix. After 13 days Chloramphenicol and Mix exposure, the microbial diversities increased slightly but remained lower than the diversities of Control and Kanamycin treatment groups, which remained high throughout the experiment (Fig. 3A). This outcome corresponds to the well diffusion test results. Plates with water containing chloramphenicol (Chloramphenicol and Mix) showed clear zones of inhibition, which were absent in the other treatment groups (Kanamycin and Control) (Supplementary Fig. 2). The alpha diversity of the female ovipositor microbiota (Mixed ANOVA on Shannon diversity: treatment: *p* = 0.033; time: *p* < 0.001; time x treatment: *p* < 0.001) (Supplementary Fig. 3) and the gut microbiota was similarly influenced (Mixed ANOVA Type III on Shannon diversity: treatment: *p* = 0.276; time: *p* < 0.001; time x treatment: *p* < 0.001) (Supplementary Fig. 4), which underlines the effectiveness of our chosen treatment (Complete dataset: supplementary spreadsheet 2).

To select spike community strains for manipulating the paternal sex-specific microbiota (experiment 3), we validated their successfully depletion by the antibiotic treatments. Five bacterial strains (Paternal: *Kiloniella*, *Marinomonas*; Maternal: *Sulfitobacter*; Environmental/ubiquitous: *Shewanella*, *Vibrio)* were depletable by chloramphenicol and could not be detected after 192 h (Fig. 3B-F). *Pseudoalteromonas*, initially handled as a potential candidate for paternal vertical transfer, could not be depleted by chloramphenicol treatment and was therefore excluded from the spike community (Supplementary Fig. 5).

### Impact of antibiotics on microbial community structure (beta diversity)

Similar to alpha-diversity, a repeated measure PERMANOVA suggested significant differences in treatment, time and the treatment x time interaction (all *p* < 0.001) of the male brood pouch microbiota composition (beta-diversity, Supplementary Table 3).

**Fig. 4:**
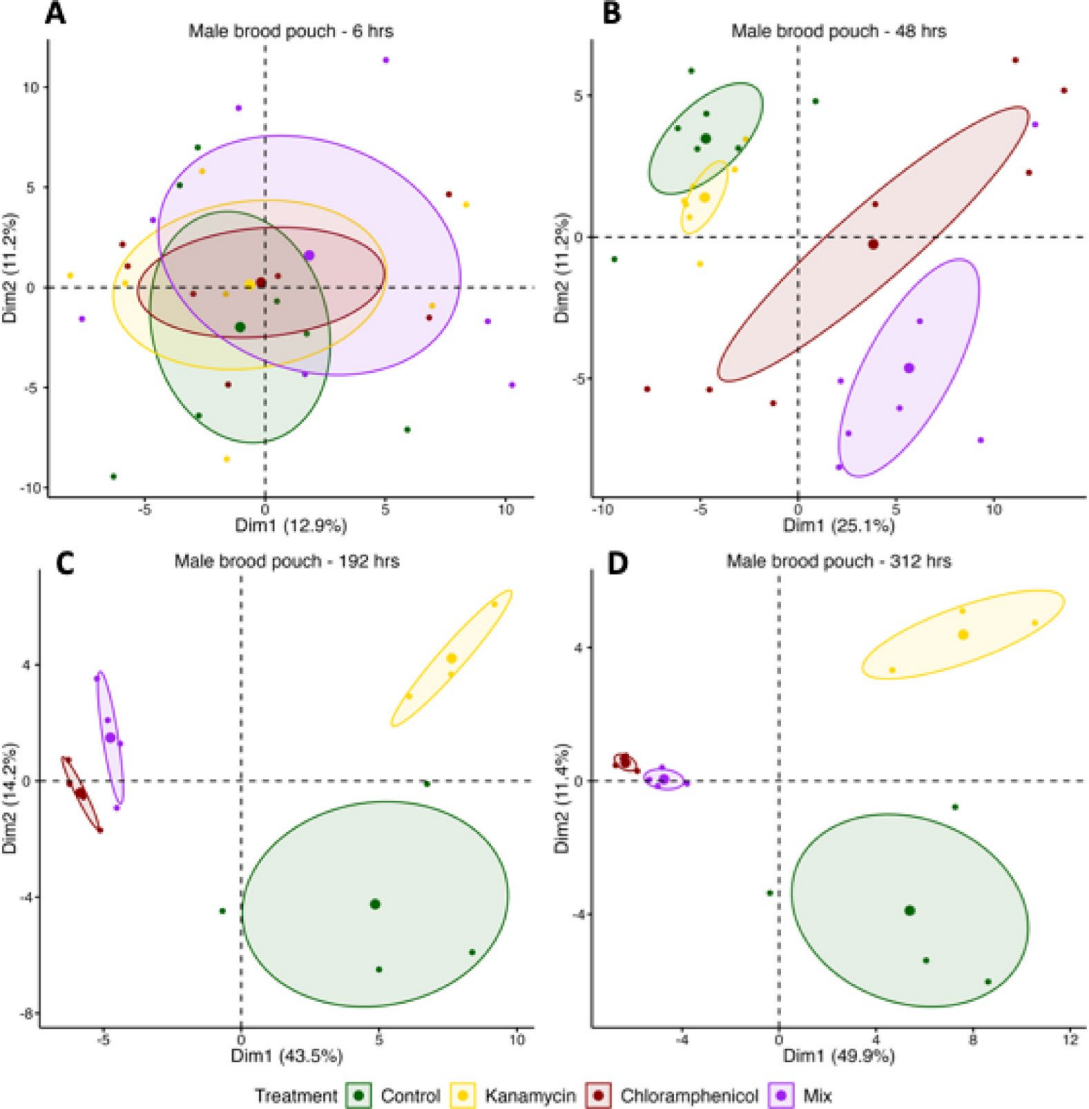
Principal component analyses (PCAs) showing the effects of antibiotic treatment on the microbiota of the male brood pouch at different time points. Panels A and B (6 h and 48 h) represent the treatment phase, while panels C and D (192 h and 312 h) show the maintenance phase. PCAs are based on normalized, log-transformed ASV data (ASVs present in > 20% of samples). Colors indicate treatment groups: Control (green), Kanamycin (yellow), Chloramphenicol (red), and Mix (purple). Ellipses represent 95% confidence intervals around the group centroid.

Principal component analyses (PCA) of four time points (treatment: 6, 48 h; maintenance: 192, 312 h) suggested a similar male brood pouch microbiota composition in all treatments at the onset of the antibiotic application (MANOVA: T6: *p* = 0.749, Fig. 4A). After 48 h microbial communities separated into Chloramphenicol and Mix treated individuals vs. Kanamycin treated and control individuals (T48: *p* = 4.96e-07, Fig. 4B). This separation intensified over time and was strongest after 192 h (T192: *p* < 2.854e-07, T312: *p* < 4.374e-05; Fig. 4C-D). Similar shifts were observed in the female ovipositor microbiota (Supplementary Fig. 7).

An indicator species analysis for T192 displayed associations between species patterns and combinations of groups, the 30 strains with the highest contribution to the observed patterns are displayed (Supplementary Fig. 6). At T192, the Chloramphenicol-treated bacterial composition was driven by *Paraglaciecola* and *Maribacter*, whereas Kanamycin and Control-composition were driven by *Sulfitobacter*,* Portibacter* and *Pseudoalteromonas* (Complete dataset: supplementary spreadsheet 3).

The antibiotic application modified the bacterial composition and reduced its diversity. Chloramphenicol was suitable to deplete a large number of strains within the characterized microbiota and depleted the five isolates chosen as spike community for experiment 3. 192 h antibiotic treatment appeared most suitable for experiment 3, minimizing stress and disease risk as opposed to longer treatments.

### Impact of antibiotic and Spike treatments on pregnancy and parental tissues

**Fig. 5:**
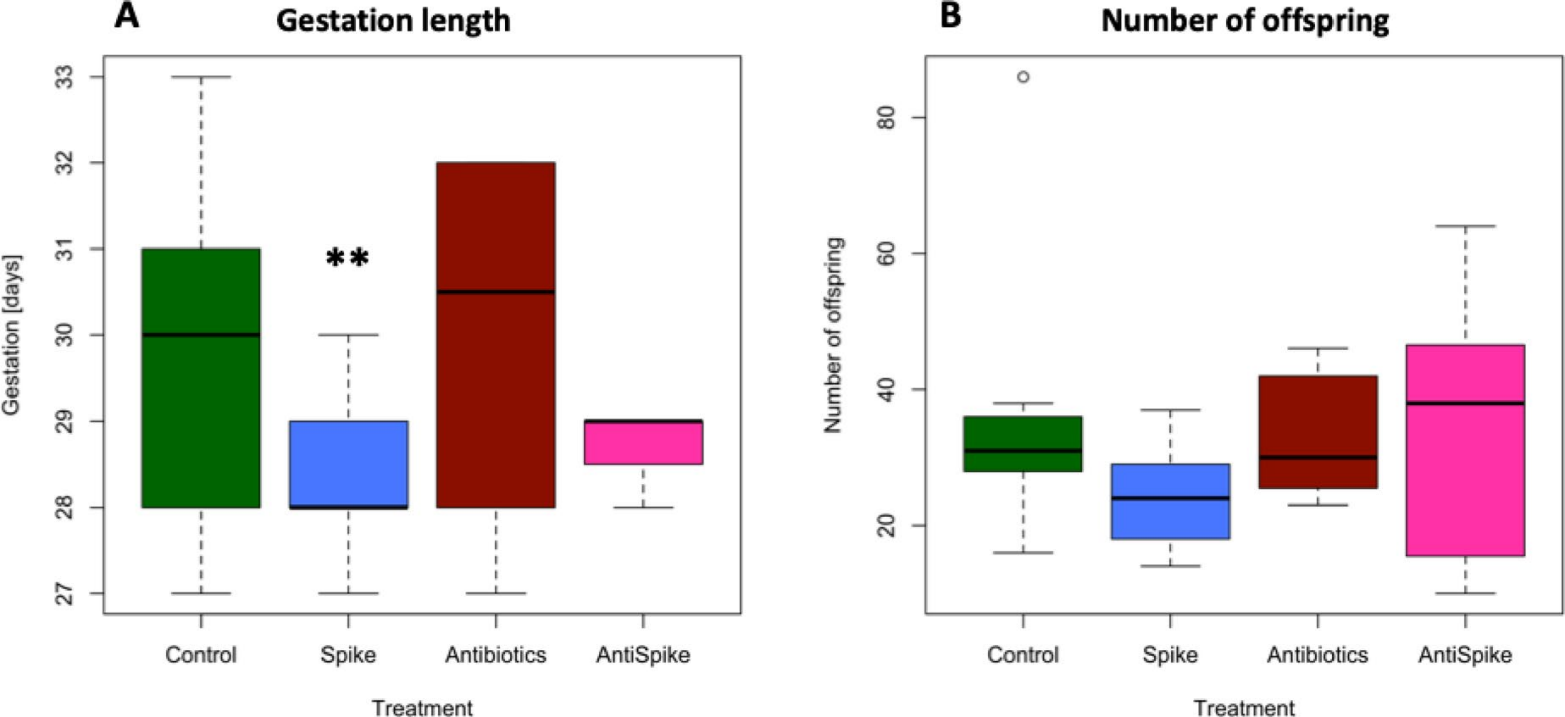
Gestation length (days) (**A**) is significantly shorter for males who received a spike treatment. In contrast, number of offspring (**B**) does not differ between males from different treatment groups. Asterisks indicate significant difference of Spike vs. all other treatments (ANOVA; *p* = 0.01).

Experiment 3 aimed to assess the impact of paternal antibiotic and spike treatment on pregnancy, early microbial colonization and offspring health from mating until 45 days post release (dpr). Male pipefish were pregnant for 29.18 ± 1.62 days (mean + SD), while the spike treatment reduced the length of gestation for approximately one day (28.47 ± 0.8 days) (SP: *p* = 0.01). The proportion of brood pouch filled with eggs and number of offspring born was not significantly influenced by any of the treatments (Fig. 5, Supplementary Table 4) (Complete dataset: supplementary spreadsheet 4).

**Fig. 6:**
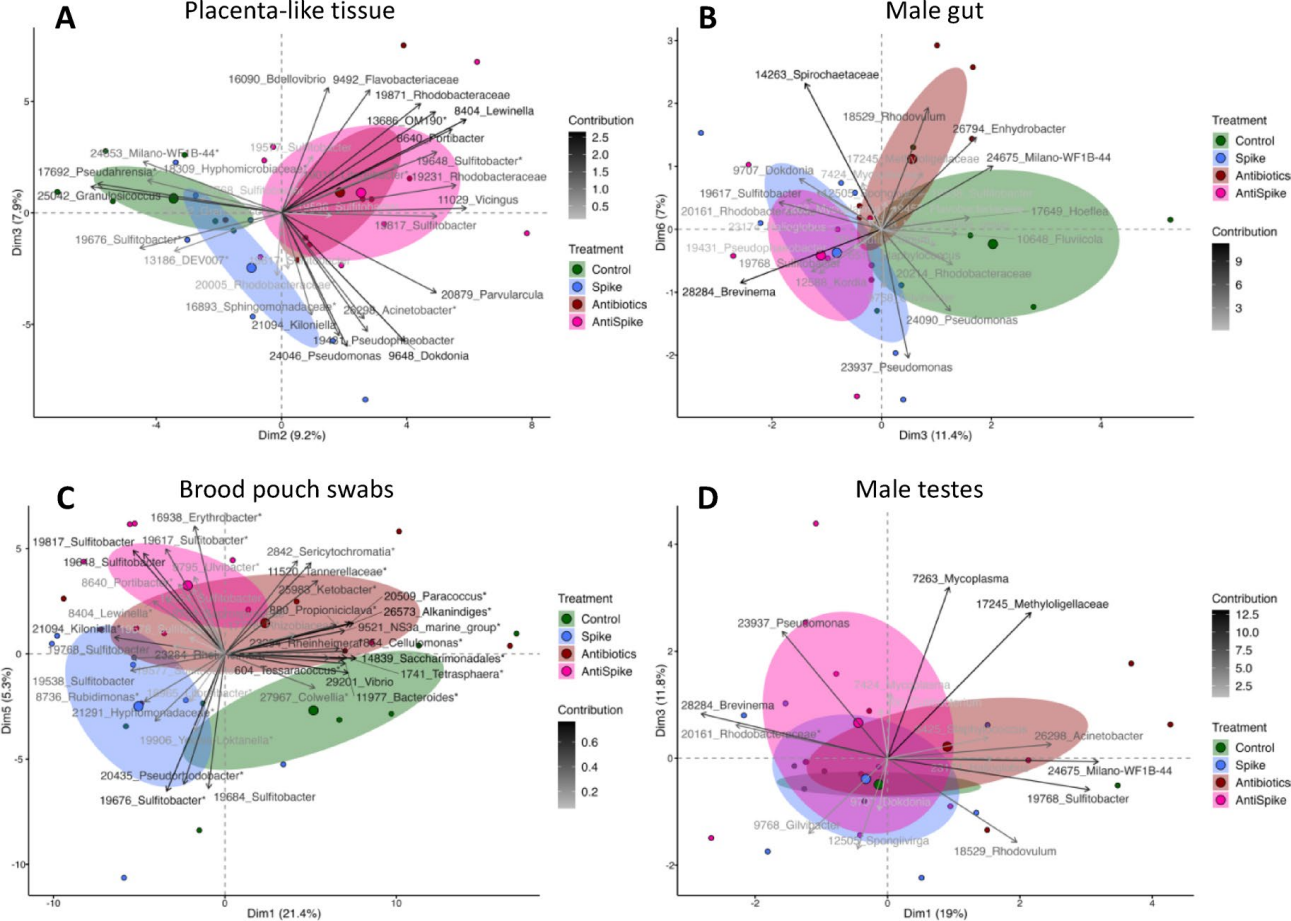
PCA biplots displaying treatment effects (antibiotics and spike) on paternal tissue. In the placenta-like tissue (**A**) and on the surface of the brood pouch (**B**), antibiotics affected the microbial composition. In the male gut (**C**) the spike treatment had a significant impact whereas in the testes (D), none of the treatments showed an effect. Colours represent the paternal treatment group: green = Control, blue = Spike, red = Antibiotics, pink = AntiSpike, Ellipses represent 95% confidence intervals around the group centroid. Factor map shows ASV ID and genus name respectively family name.

For microbiota sequencing, a total of 1080 samples, including adult tissue, juvenile tissue and water samples, were 16 S rRNA MiSeq amplicon sequenced. 34,021,625 reads were returned (mean run 63: 29,912 reads/sample, mean run 64: 27,566.5 reads/sample, mean run 65: 23,589 reads/sample, mean run 79: 135,136.5 reads/sample), along with 23,841 representative sequences (Supplementary Table 1). Male pipefish were sampled after giving birth (approximately day 30 post-treatment). We analysed alpha diversity (Shannon) and beta diversity of the placenta-like tissue microbiome and its surface. Alpha diversity of the males was not influenced by treatment (Supplementary Fig. 9, Supplementary Table 5). Antibiotic treated fish (Antibiotics and AntiSpike group) showed a significantly different microbial composition (beta diversity, *p* = 0.002) on the surface (brood pouch swabs) and within the placenta-like tissue (Fig. 6A and C) compared to non-antibiotic treated fish (Control and Spike) (Fig. 6A and C). Male gut microbiome was not influenced by the antibiotic treatment (Fig. 6B). In contrast, the Spike group overlapped with the AntiSpike group and differed from the other groups (SP: *p* = 0.027). In the testes, all groups overlapped in their microbiome and no statistical differences were identified (Fig. 6D). While males were sampled after giving birth, females (all untreated) were sampled directly after mating. Sequencing the female gut and ovipositor microbiota allowed (a) to illuminate sexual dimorphism in gut microbiome when comparing to gut microbiome of untreated males, and (b) provided a positive control for the presence of our spike community, as the ovipositor of a mated female had been in direct contact with the spike-treated male brood pouch when mated with males from AntiSpike and Spike treatment groups (treatment assignment thus refers to the treatment of the male mating partner).

Treatment had no detectable effect on female pipefish microbiomes in terms of alpha diversity, beta diversity, or spike community detection (Supplementary Figs. 10–11).

Sex was an important factor explaining variation in alpha (*p* = 0.0002, Supplementary Fig. 13, Supplementary Table 6) and beta diversity of gut microbial composition (*p* = 0.026, Supplementary Fig. 12, Supplementary Table 7), suggesting a sexual microbiome dimorphism in untreated pipefish. In the gut tissue, this dimorphism was driven by the indicator species *Aliivibrio* in the female gut as well as *Rhodobacteriaceae*, *Maribacter*, *Enhydrobacter* and *Milano-WF1B-44* in the male gut (Supplementary Fig. 12) (Complete dataset: supplementary spreadsheet 5).

### Impact of antibiotic and Spike treatments on life-history and offspring Microbiome

**Fig. 7:**
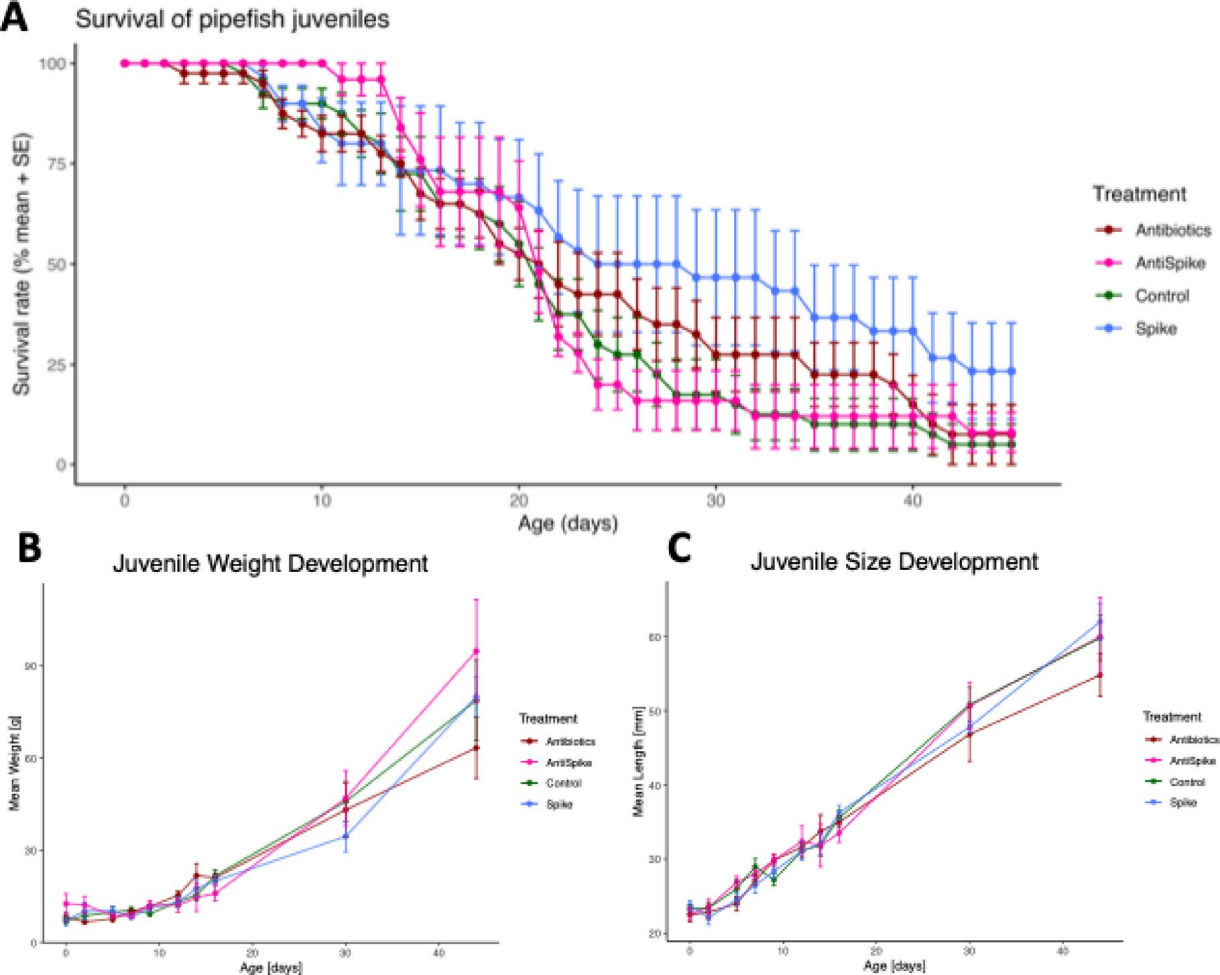
Life-history data of pipefish juveniles from birth until 45 days after birth. Survival rate (**A**), Weight development (**B**) and Size development (**C**). All plots show mean ± standard error (SE). Colour represents paternal treatment.

Offspring weight was influenced by time supporting a weight gain throughout development (time: *p* < 2e-16), but not by treatment or the interaction of treatment and time (treatment: *p* = 0.80; treatment*time: *p* = 0.24) (Fig. 7B, Supplementary Table 8). Offspring size was influenced by time and its interaction with treatment (time: *p* < 2e-16; time*treatment: *p* = 0.045), while treatment alone had no significant effect (*p* = 0.17) (Fig. 7C, Supplementary Table 9). Treatment, time and their interaction significantly affected offspring survival (treatment: *p* = 1.85e-14; time: *p* < 2e-16; treatment*time: *p* = 8.03e-10) (Supplementary Table 10). Offspring of spike-treated fathers showed significantly higher overall survival across the time course compared to all other groups (Contrasts from the full model: Spike vs. Antibiotics, AntiSpike, and Control: all *p* < 0.0001; Fig. 7A, Supplementary Table 11) (Complete dataset: supplementary spreadsheet 4). Even though paternal spike treatment enhanced offspring survival, overall juvenile mortality rate was high throughout the experiment, which is consistent with natural mortality rates observed in syngnathids. After leaving the protective paternal brood pouch, only a small fraction of offspring typically survives to adulthood due to predation and influences of environmental factors^27^.

**Fig. 8:**
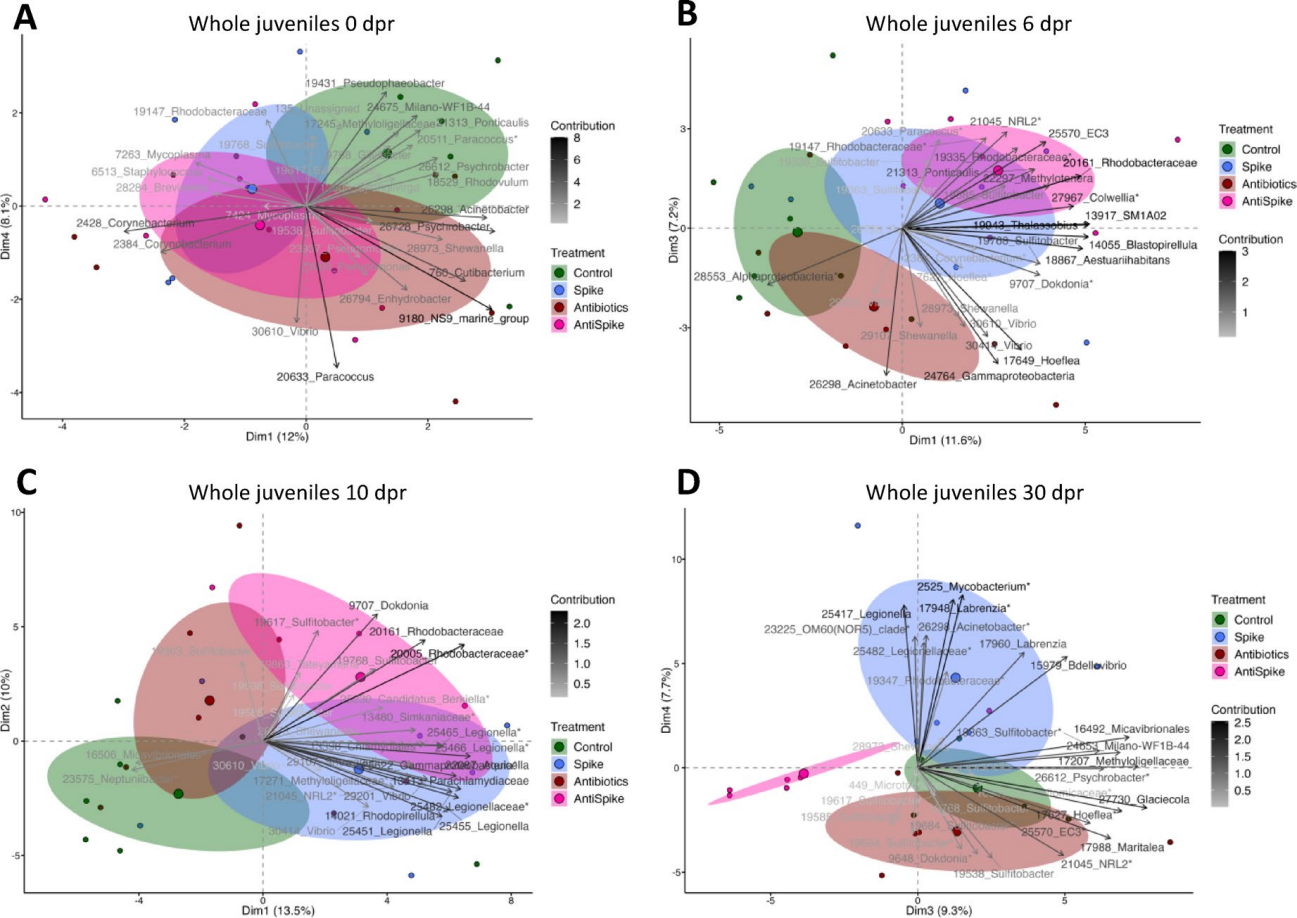
Shifts in offspring whole body microbiome composition from birth (**A**), 6 days (**B**), 10 days (**C**) up to 30 days (**D**) after birth (dpr = days post-release). Colours represent the paternal treatment group. Ellipses represent 95% confidence intervals around the group centroid. Factor map shows ASV ID and genus name respectively family name.

Upon offspring birth, juvenile whole-body and gut microbiomes were sequenced every second day for two weeks, after 30 and 45 days. Offspring from untreated fathers (Control) had a lower whole-body microbiota diversity than offspring from treated fathers across all time points (Alpha diversity – factor treatment: *p* = 0.039), while the diversity of the microbiome did not change over time (*p* = 0.57, Supplementary Fig. 13 A, Supplementary Table 12). Conversely, offspring gut microbiome changed (Alpha diversity) over time (*p* < 0.001), but was unaffected by the paternal treatment (Supplementary Fig. 13B, Supplementary Table 13). At birth, offspring whole-body microbiome was unaffected by paternal treatment (dpr 0). However, two days after birth, the spike treatment significantly shaped offspring microbiome (*p* = 0.013), while paternal antibiotics treatment indicated a trend (*p* = 0.052). Paternal treatment effects were strongest between six and ten days after birth (antibiotics: dpr 6: *p* = 0.039; dpr 10: *p* = 0.025) and (spike: dpr 6: *p* = 0.006; dpr 10: *p* = 0.017), but remained detectable up to 30 days after birth (antibiotics: *p* = 0.025; spike: *p* = 0.009) (Supplementary Table 14, Fig. 8). The absence of a treatment effect at dpr 8 and dpr 12 indicates fluctuations in paternal treatment effects.

Paternal spike treatment did not shape offspring gut microbiome, with the exception of 12 days post-release (*p* = 0.044; Supplementary Table 15, Supplementary Fig. 14) (Complete dataset: supplementary spreadsheet 6 and 7). Throughout the sampling period, microbial communities in seawater and Artemia clustered separately from the juvenile fish microbiota, indicating distinct community compositions across sample types (Supplementary Fig. 16).

### Detection and persistence of Spike community bacteria in adult and offspring microbiomes

**Fig. 9:**
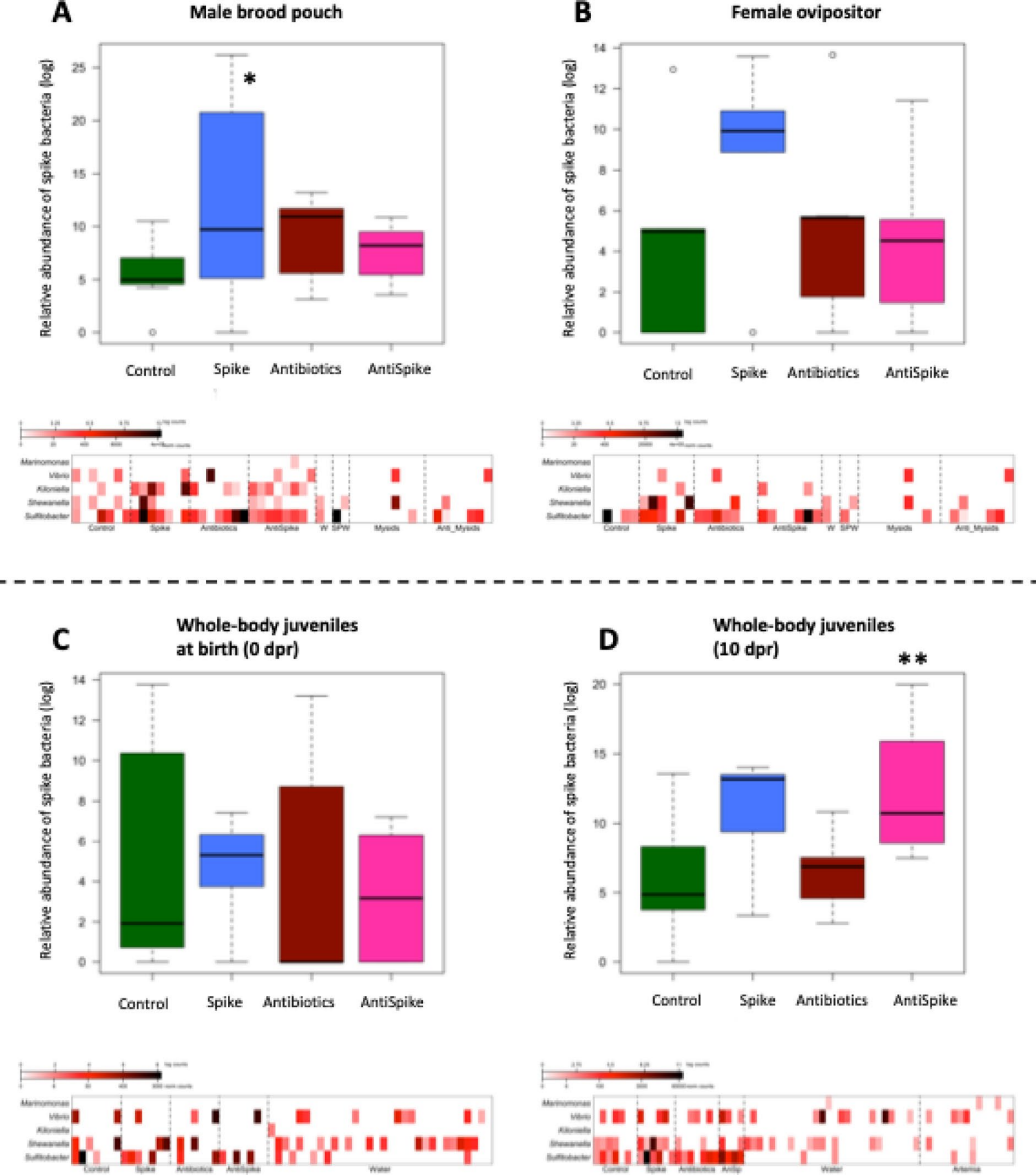
Prevalence of spike bacteria. Upper boxplots show the relative abundance (log scale) of spike bacteria within the whole microbiome of the respective tissue. Colours represent the paternal treatment group. Lower heatmaps display normalized individual sample counts; darker red indicates higher bacterial abundance. **A**: Male brood pouch surface swabs. **B**: Female ovipositor after contact with treated male brood pouch. **C**: Whole-body juveniles at birth (0 dpr). **D**: Whole-body juveniles 10 days after birth (10 dpr). In the heatmaps, samples are grouped by treatment in the following order (left to right): Control, Spike, Antibiotics, AntiSpike, followed by environmental sources where applicable: Water (W), Spike Water (SPW), Mysids, and Antibiotic-treated Mysids (Anti_Mysids).

When examining the 16 S rRNA sequences of our spike community in the relevant tissues after approximately two (females) respectively 30 days of pregnancy (males), we found identical sequence hits across all tissues (both adults and juveniles) and treatment groups, underlying that the microbes used in our spike community are members of the natural pipefish microbiome. In tissue of spike-treated fathers (Spike and AntiSpike), relative abundances of spike community members were undistinguishable from untreated fathers (Control and Antibiotics) (Fig. 9A, Supplementary Fig. 15). Normalized counts of Spike members were higher on the surface of male brood pouches (brood pouch swabs) from spike treated fathers (*p* = 0.044), a pattern driven by high *Shewanella* prevalence (*p* = 0.018). However, across all adult tissues (male brood pouch and female ovipositor) and treatments *Shewanella* counts were high, while *Marinomonas* was mostly absent indicating a lack of colonization (Fig. 9A-B, Supplementary Table 16).

At birth, spike bacteria were already detectable in the whole-body microbiome of juveniles across treatments (Fig. 9C), although with no significant treatment differences. Paternal spike treatment (spike and AntiSpike) induced spike community prevalence in offspring whole-body microbiome at 10 days after birth (*p* = 0.013), driven by high counts of *Shewanella* (*p* = 0.018), particularly in the AntiSpike treatment group (Fig. 9D). No similar tendency was identified for offspring gut microbiome. *Kiloniella* was absent from juvenile gut samples, suggesting a lack of transfer respectively colonization. Regardless of paternal treatment, *Marinomonas* was absent in the majority of offspring samples (both whole-body-microbiome and gut-microbiome), but prevalent in the microbiome of the food (*artemia*, Fig. 9D). Since this strain did not colonize adult tissue (Supplementary Table 16), the rare counts for this strain in our offspring dataset probably stem from food or other environmental influences. *Vibrio* counts in the offspring should be treated with caution, as a ubiquitous strain in the natural environment of the pipefish, *Vibrio* was particularly abundant in artemia, the food of the juveniles (Complete dataset: supplementary spreadsheet 8).

## Discussion

We aimed to experimentally assess the existence and role of paternal microbiota provisioning on offspring life-history and early microbial colonization through male pregnancy in the sex-role reversed pipefish *Syngnathus typhle*. To achieve this goal, we cultivated and characterized the sex-specific pipefish microbiota (experiment 1) and studied the effectiveness of antibiotics in depleting its microbiomes (experiment 2). This facilitated to select microbes for paternal microbial manipulation (experiment 3) to unravel how paternal microbial provisioning influenced offspring development and microbiome establishment.

Both cultivation-dependent and cultivation-independent approaches^12^ supported the presence of distinct microbial communities in female eggs, the brood pouch of non-pregnant males, and throughout male pregnancy. Early offspring microbial colonization was tissue-specific, shaped rather independently by mother and father^8^.

The here observed sexual dimorphism in microbial communities, coupled with a decreased beta diversity in females (Supplementary Fig. 11, 12), strikingly contrasts with findings from conventional sex role species^28,29^. Sex roles and life history, rather than sex alone, seem to drive immunological activity and microbial diversity^30,31^. The differences in investment into parental care and secondary sexual ornaments appear to extend into the complex interactions between the microbiome and the immune system. Investigating parental microbial provisioning will help to understand how sex-role dynamics influence health and development across generations.

By treating pipefish with chloramphenicol, we successfully decreased pipefish brood pouch microbial diversity. Chloramphenicol was particularly effective against the microbes later assigned as experimental spike community, consisting of microbes from parental origin and isolates ubiquitous in pipefish and their natural environment. Achieving a completely germ-free status in our fish remained unattainable with antibiotic treatment alone. Working with *S. typhle*, a non-model marine species, presents challenges for gnotobiotic rearing due to its obligatory male pregnancy and the largely unknown impact of environmental microbes on its development and health. We thus acknowledge the inherent bias of persistent microbes, that we were unable to exclude throughout the conducted experiments.

Treating paternal pipefish with antibiotics influenced the composition and diversity of their microbiomes. The antibiotic treatment effect remained detectable 30 days post-treatment (after male pregnancy) when assessing the microbiome of the placenta-like tissue and its surface, but was absent when assessing the microbiome of the male gut or testes. This suggests that the antibiotic treatment affected the parts of the body, which were in direct contact with the water, and penetrated the underlying tissues, while it did not have a long-lasting effect on internal organs. After the antibiotic treatment, the gut microbiome might have reverted towards a stable core microbiome during the four weeks of pregnancy. In contrast, a long-lasting influence of the spike treatment on the microbiome composition was observed in the male gut despite that bacteria were added directly into the brood pouch. To shed light on time dynamics of potentially interacting antibiotic and spike treatments on parental tissues, sequencing the microbial composition of paternal tissue throughout male pregnancy would be necessary. We have decided against this approach in the concurrent experiment to avoid disturbing or sacrificing the developing embryos due to limited housing opportunity in our experiment (36 breeding pairs in separate tanks, 9 per treatment).

Paternal spike treatment shortened paternal pregnancies compared to those of unexposed or antibiotic-treated fathers (Control, Antibiotics and AntiSpike). Also, in mammalian pregnancy various types of bacteria have been linked to preterm labour^32^. However, contrary to health challenges observed upon mammalian preterm birth, offspring from spike-treated fathers survived better than those from all other groups, suggesting spike treatment to be adaptive for offspring health.

After offspring left the paternal brood pouch and the associated protection from environmental microbial colonization, paternal spike as well as antibiotic treatment started to influence the whole-body microbiome up to 30 days after birth (Fig. 8B-D, Supplementary Table 14). This suggests that the shift in the paternal brood pouch microbiome upon paternal treatment must have influenced the offspring microbial composition and the susceptibility of the offspring towards microbial colonization. The latter could explain why paternal treatment effects only emerged after birth, when offspring were suddenly exposed to a cocktail of surrounding environmental bacteria. In contrast, the offspring gut microbiome was not influenced by paternal treatment. While an impact on offspring gut microbiome by paternal treatment was potentially masked by a strong influence of the bacteria consumed over artemia feeding, this finding supports the notion that pipefish fathers primarily shape the whole-body microbiome, while mothers shape the gut microbiome^8^.

Despite initiating the spike community treatment with a mixture of pure bacterial cultures (Supplementary Fig. 8), sequences matching our spike bacteria were found across all tissues (adults and juveniles) and treatment groups. This either suggests that the respective bacteria also colonized through environmental sources, making experimentally applied and natural strains difficult to detect. Or, non-mutually exclusive, that the sequences obtained from 16 S V3/V4 ribotyping were too conserved to reliably detect the specific strains provided in the spike community. In particular *Shewanella* and *Vibrio* often exhibit high 16 S rRNA copy numbers, leading to taxonomic classification differences for the same strain^33^. Metagenomics could have better distinguished bacterial strains, however, the high sample size sequenced throughout the experiment (*n* = 1080), rendered this impossible.

Although this experiment focused solely on paternal microbial transmission, the selected bacteria originated from paternal, maternal, and environmental sources, allowing us to assess microbial specificity in transgenerational effects. *Sulfitobacter*, identified as maternally specific in experiment 1, exhibited high prevalence upon inoculation (Spike and AntiSpike) in paternal tissue post-pregnancy and offspring tissues. Its presence, even in lower counts in Control and Antibiotic-treated groups, suggests that it was environmentally ubiquitous, allowing horizontal transfer after antibiotic effects ceased. Conversely, *Marinomonas*, previously identified as paternal-specific in all studies on *S. typhle* microbiota, was found only in low abundances on the brood pouch surface within the AntiSpike group and was absent in all offspring tissues. Similarly, *Kiloniella*, which was present in high abundances in the paternal placenta-like tissue upon spike exposure, could not be detected in offspring. It is tempting to speculate that while these isolates are naturally sex-specific, they were outcompeted by other isolates of maternal or environmental origin upon paternal inoculation. Several ecological, physiological, and methodological factors may explain this outcome. Successful microbial colonization depends on complex host-microbe and microbe-microbe interactions. *Marinomonas* may have lacked a competitive advantage within the juvenile microbiome or required specific host-derived factors that were absent in this experimental setting. Additionally, colonization success might be influenced by resilience to environmental shifts, or the ability to utilize host-associated nutrients, which could have been suboptimal for *Marinomonas* in this context. Finally, methodological constraints, such as differences in initial inoculation density or detection biases in our sequencing pipeline, may have contributed to its absence. Future studies employing strain-specific tracking methods, such as qPCR or fluorescence tagging, could help clarify whether *Marinomonas* and *Kiloniella* were present but remained below the detection limit, or if they truly failed to establish within the microbiota.

In line with earlier studies, paternal spike treatment only shaped the whole-body microbiome^8^. This underlines the importance in disentangling multiple routes of vertical bacteria transfer, as maternal transfer was previously suggested to rather shape offspring gut microbiomes^8^giving both parents specific roles in microbial inheritance. In natural systems such as *S. typhle*, differentiating between environmental and parental effects on microbial colonization and development is essential. The interplay between these factors is complex and requires comprehensive analysis to fully understand the patterns involved. Due to the discussed limitations of our approach, future research should apply genetic-fluorescent- and antibiotic resistance-tagged spike strains. This would facilitate a detailed exploration of microbial transfer pathways from parents to offspring, providing precise insights into how specific bacteria are transmitted and established across generations, as well as pinpointing their functions in both paternal as well as offspring microbiomes.

By assessing the parental as well as offspring immune responses upon both antibiotic and spike exposures, we could gain deeper insights into the complex interactions between microbial communities, environmental factors, and host immune systems across generations, linking to previously suggested bi-parental trans-generational immune priming^34–36^. Paternal spike bacteria not only shaped offspring microbiomes but also increased offspring survival at a larger body size, highlighting the potential adaptive significance of paternal microbial transfer. While our analysis focused on relative abundances to assess shifts in microbial composition, incorporating absolute abundances could provide additional insights into microbial dynamics. Quantifying total bacterial loads would help determine whether observed differences stem from actual changes in microbial colonization or simple shifts in relative proportions. This distinction could be particularly relevant for evaluating microbial depletion efficacy and the extent of paternal microbial transfer. However, absolute quantification requires additional techniques such as qPCR^37^which were beyond the scope of this study. Future research integrating these methods will refine our understanding of microbial transmission in pipefish and its ecological and evolutionary implications.

## Conclusion

Our study underscores the significant role of both paternal and environmental factors in shaping the microbiota of offspring. It also highlights the necessity for advanced methodologies and comprehensive studies to unravel the complexities of microbial transfer and its implications for host health. Continued research in this area will not only enhance our understanding of microbial ecology but may also contribute to managing microbial communities for promoting health and preventing disease.

This work highlights the role of paternal provisioning during male pregnancy, demonstrating that early colonization patterns in pipefish juveniles are primarily tissue- rather than sex- or bacterial strain-specific. This indicates that a dysbiosis in the father’s sex-specific tissues may notably influence offspring microbial colonization, as demonstrated here for the maternal indicator *Sulfitobacter*. While these findings might slip our attention in a balanced ecosystem, they could gain significance in the context of global change, potentially leading to the transfer of an altered microbial community with unknown impacts on offspring health and development. Our study examines only a small part of a complex and dynamic system, and the full impact of various microbial sources, including environmental bacteria, remains to be predicted in future studies. This underscores the need for advanced methodologies, such as fluorescent tagging, to better understand microbial transfer pathways in more detail. Future research should focus on exploring these transfer mechanisms and their long-term effects on offspring health to provide a more comprehensive understanding of the ecological and evolutionary implications of microbial inheritance.

## Supplementary Information

Below is the link to the electronic supplementary material.

Supplementary Material 1

Supplementary Material 2

Supplementary Material 3

Supplementary Material 4

Supplementary Material 5

Supplementary Material 6

Supplementary Material 7

Supplementary Material 8

Supplementary Material 9

Supplementary Material 10

## Data Availability

Sequencing data files of the manuscript have been deposited on the Mendeley Data Server and can be accessed via 10.17632/2t2vvrcm6g.3 (Experiments 1 and 2) and 10.17632/d29×97ds3.1 (Experiment 3).
