# Supplementary material for: Paternal microbiota manipulation influences offspring microbial colonization and development in a sex role-reversed pipefish
Source: Sci Rep. 2025 Aug 22;15:30911. doi: 10.1038/s41598-025-16222-y (PMC12373867; doi:10.1038/s41598-025-16222-y)
Supplement: Supplementary file 1 — Supplementary Material 1 [file 41598_2025_16222_MOESM1_ESM.docx]

Paternal microbiota manipulation influences offspring microbial colonization and development in a sex role-reversed pipefish

Kim-Sara Wagner, Frédéric Salasc, Silke-Mareike Marten & Olivia Roth

Supplementary Tables:

| **MiSeq Run** | 47 | 48 | 63 | 64 | 65 | 79 |
| --- | --- | --- | --- | --- | --- | --- |
| **Experiment** | Experiment 2 | | Experiment 3 | | | |
| **Samples** | 377 | 377 | 289 | 384 | 377 | 30 |
| **Min. reads** | 22 | 22 | 13 | 7 | 16 | 57639 |
| **Median** | 28517.0 | 21022.0 | 29912.0 | 27566.5 | 23589.0 | 135136.5 |
| **Mean** | 30871.65 | 21747.52 | 30129.50 | 28620.82 | 26122.26 | 149190.37 |
| **Max. reads** | 102427 | 55732 | 83458 | 115401 | 137303 | 344546 |
| **Total reads** | 11638610 | 8198814 | 8707426 | 10990395 | 9848093 | 4475711 |
| **Rep seqs** | 10688 | | 23841 | | | |

Supplementary Table 1: QIIME2 sequence processing statistics. Samples were randomly distributed over all sequencing runs. DADA2 workflow was applied separately for each run before merging all runs into a master data set.

**Experiment 1: Cultivation and characterization of sex-specific microbiota**

| **Pairs** | **Df** | **SumsOfSqs** | **F.Model** | **R2** | **p.value** | **p.adjusted** |
| --- | --- | --- | --- | --- | --- | --- |
| FG - NP | 1 | 0.6658655 | 1.4589270 | 0.05126430 | 0.035 | 0.1050000 |
| FG - EPP | 1 | 0.5352662 | 1.1910171 | 0.05367113 | 0.176 | 0.3016667 |
| FG - EPJ | 1 | 0.4758822 | 1.0656973 | 0.04829656 | 0.369 | 0.4612500 |
| FG - LPP | 1 | 0.5355834 | 1.1764959 | 0.04866279 | 0.167 | 0.3016667 |
| FG - LPJ | 1 | 0.7346852 | 1.6352001 | 0.06637657 | 0.004 | **0.0200000** |
| NP - EPP | 1 | 0.3875046 | 0.8660220 | 0.03482752 | 0.689 | 0.7382143 |
| NP - EPJ | 1 | 0.5451620 | 1.2252517 | 0.04857243 | 0.181 | 0.3016667 |
| NP - LPP | 1 | 0.5849061 | 1.2918955 | 0.04733623 | 0.087 | 0.2175000 |
| NP - LPJ | 1 | 0.9318041 | 2.0822734 | 0.07414903 | 0.001 | **0.0150000** |
| EPP - EPJ | 1 | 0.3080231 | 0.7114310 | 0.03802120 | 0.891 | 0.8910000 |
| EPP - LPP | 1 | 0.4249862 | 0.9564965 | 0.04564200 | 0.540 | 0.6230769 |
| EPP - LPJ | 1 | 0.8262708 | 1.8886978 | 0.08628644 | 0.002 | **0.0150000** |
| EPJ - LPP | 1 | 0.4849914 | 1.0990119 | 0.05208831 | 0.328 | 0.4612500 |
| EPJ - LPJ | 1 | 0.7400999 | 1.7034783 | 0.07848872 | 0.006 | **0.0225000** |
| LPP - LPJ | 1 | 0.4841525 | 1.0888442 | 0.04715889 | 0.354 | 0.4612500 |

Supplementary Table 2: Pairwise adonis comparing sexes and/or stages of pregnancy in S. typhle. Abbreviations: FG = female gonads, NP = non pregnant, EPP = early pregnant pouch, EPJ = early pregnant juveniles, LPP = late pregnant pouch, LPJ = late pregnant juveniles.

**Experiment 2: Effectiveness of antibiotics for natural pipefish microbiota depletion**

|  | **Df** | **SumOfSqs** | **MeanSqs** | **F.Model** | **R2** | **Pr(>F)** |
| --- | --- | --- | --- | --- | --- | --- |
| **Treatment** | 3 | 7.885 | 2.6282 | 13.308 | 0.06948 | 0.001 *** |
| **Time** | 1 | 7.962 | 7.9623 | 40.317 | 0.07016 | 0.001 *** |
| **Individual** | 52 | 33.077 | 0.6361 | 3.221 | 0.29146 | 0.001 *** |
| **Treatment:Time** | 3 | 8.87 | 2.9568 | 14.972 | 0.07816 | 0.001 *** |
| **Residuals** | 282 | 55.692 | 0.1975 |  | 0.49074 |  |
| **Total** | 341 | 113.486 |  |  | 1 |  |

Supplementary Table 3: Repeated measures PERMANOVA reveals significant impact of time, treatment and individual on microbial beta-diversity of the sex-specific microbiota

**Experiment 3: Manipulating paternal sex-specific microbiota to unravel impact on the offspring**

**Experiment 3 (A) – Adults**

| **Treatment** | **Filling of brood pouch**  **(% + SD)** | **Gestation length**  **(mean days + SD)** | **Number of offspring**  **(mean + SD)** |
| --- | --- | --- | --- |
| **All fish** | 86.76 ± 18.7 | 29.18 ± 1.62 | 31.76 ± 15.06 |
| **Spike_Treat** | 85.3 ± 21.76 | 28.47± 0.8 | 28.88 ± 14.69 |
| **Anti_Treat** | 90.63 ± 15.48 | 29.38 ± 1.63 | 33.63 ± 14.41 |
| **Control** | 91.67 ± 12.5 | 29.78 ± 1.86 | 36.11 ± 19.77 |
| **Spike** | 75 ± 25 | 28.22 ± 0.97 | 24.11 ± 7.85 |
| **Antibiotics** | 84.38 ± 18.6 | 30 ± 2.14 | 33 ± 9.12 |
| **AntiSpike** | 96.86 ± 8.84 | 28.75 ± 0.46 | 34.25 ± 18.99 |

Supplementary Table 4: Pregnancy statistic for all treatment groups. Spike_Treat includes fish of the treatment groups Spike and AntiSpike and Anti_Treat includes fish of the groups Antibiotics and AntiSpike.

|  | Df | Sum Sq | Mean Sq | F value | Pr(>F) |
| --- | --- | --- | --- | --- | --- |
| Organ | 3 | 69.02 | 23.005 | 28.424 | **3.32e-13 ***** |
| Treatment | 3 | 3.69 | 1.230 | 1.520 | 0.214 |
| Organ:Treatment | 9 | 10.41 | 1.156 | 1.429 | 0.187 |
| Residuals | 95 | 76.89 | 0.809 |  |  |

Supplementary Table 5: Two-way ANOVA comparing male organs under the influence of different treatments.

|  | **Df** | **Sum Sq** | **Mean Sq** | **F value** | **Pr(>F)** |
| --- | --- | --- | --- | --- | --- |
| Sex | 1 | 18.76 | 18.761 | 14.539 | **0.000181 ***** |
| Treatment | 3 | 3.43 | 1.142 | 0.885 | 0.449662 |
| Sex:Treatment | 3 | 1.45 | 0.485 | 0.376 | 0.770559 |
| Residuals | 206 | 265.82 | 1.290 |  |  |

Supplementary Table 6: Two-way ANOVA results suggest a sexual dimorphism within the alpha diversity of male and female pipefish

|  | **Df** | **Pillai** | **Approx. F** | **Num Df** | **Den Df** | **Pr(>F)** |
| --- | --- | --- | --- | --- | --- | --- |
| **(Intercept)** | 1 | 0.00000 | 0.00000 | 6 | 5 | 1.00000 |
| **Sex** | 1 | 0.89181 | 6.8689 | 6 | 5 | **0.02583 *** |
| **Residuals** | 10 |  |  |  |  |  |

Supplementary Table 7: One-way ANOVA results suggest a sexual dimorphism in the gut microbiota of pipefish (beta-diversity). Here, only the gut tissue of untreated males and females (Control group) was analyzed.

**Experiment 3 (B) – Juveniles**

|  | **Df** | **Sum Sq** | **Mean Sq** | **F value** | **Pr(>F)** |
| --- | --- | --- | --- | --- | --- |
| **Treatment** | 3 | 54 | 18 | 0.333 | 0.802 |
| **Time** | 1 | 16961 | 16961 | 313.608 | <2e-16 *** |
| **Treatment:Time** | 3 | 238 | 79 | 1.466 | 0.242 |
| **Residuals** | 32 | 1731 | 54 |  |  |

Supplementary Table 8: Juvenile weight development is affected by time but not by treatment

|  | **Df** | **Sum Sq** | **Mean Sq** | **F value** | **Pr(>F)** |
| --- | --- | --- | --- | --- | --- |
| **Treatment** | 3 | 7 | 2 | 1.150 | 0.3441 |
| **Time** | 1 | 4968 | 4968 | 2380.310 | **<2e-16 ***** |
| **Treatment:Time** | 3 | 19 | 6 | 2.991 | **0.0454 *** |
| **Residuals** | 32 | 67 | 2 |  |  |

Supplementary Table 9: Juvenile size development is strongly affected by time and time*treatment interaction but not by treatment alone

|  | **Df** | **Sum Sq** | **Mean Sq** | **F value** | **Pr(>F)** |
| --- | --- | --- | --- | --- | --- |
| **Treatment** | 3 | 3.31 | 1.10 | 22.95 | **1.85e-14 ***** |
| **Time** | 1 | 124.10 | 124.10 | 2583.85 | **<2e-16 ***** |
| **Treatment:Time** | 3 | 2.21 | 0.74 | 15.37 | **8.03e-10 ***** |
| **Residuals** | 1234 | 59.27 | 0.05 |  |  |

Supplementary Table 10: Juvenile survival is strongly affected by treatment, time and their interaction

| **Contrast** | **Estimate** | **SE** | **df** | **t.ratio** | **p.value** |
| --- | --- | --- | --- | --- | --- |
| **Antibiotics-AntiSpike** | 0.0230 | 0.0184 | 1234 | 1.251 | 0.5945 |
| **Antibiotics-Control** | 0.0451 | 0.0162 | 1234 | 2.792 | 0.0273 * |
| **Antibiotics-Spike** | -0.0942 | 0.0175 | 1234 | -5.398 | **<.0001 ***** |
| **AntiSpike-Control** | 0.0221 | 0.0184 | 1234 | 1.198 | 0.6283 |
| **AntiSpike-Spike** | -0.1172 | 0.0196 | 1234 | -5.992 | **<.0001 ***** |
| **Control-Spike** | -0.1393 | 0.0175 | 1234 | -7.983 | **<.0001 ***** |

Supplementary Table 11: Contrasts (pairwise, adjusted by TukeyHSD) reveal that offspring of spike-treated fathers have a significantly higher survival.

|  | **Estimate** | **Std. Error** | **t value** | **Pr(>ltl)** |
| --- | --- | --- | --- | --- |
| (Intercept) | 2.878693 | 0.128019 | 22.486 | **<2e-16 ***** |
| AntiSpike | 0.026886 | 0.163318 | 0.165 | 0.8694 |
| Control | -0.335738 | 0.161476 | -2.079 | **0.0386** * |
| Spike | -0.060166 | 0.164025 | -0.367 | 0.7141 |
| TimePoint | 0.002756 | 0.004850 | 0.568 | 0.5704 |

Supplementary Table 12: Whole-body microbiota: ANCOVA testing the influence of treatment and/or time on microbiota composition (alpha-diversity)

|  | **Estimate** | **Std. Error** | **t value** | **Pr(>ltl)** |
| --- | --- | --- | --- | --- |
| (Intercept) | 3.092842 | 0.133810 | 23.114 | **< 2e-16 ***** |
| AntiSpike | 0.127106 | 0.170633 | 0.745 | 0.457 |
| Control | -0.269765 | 0.169415 | -1.592 | 0.113 |
| Spike | -0.246375 | 0.171374 | -1.438 | 0.152 |
| TimePoint | -0.022126 | 0.004995 | -4.430 | **1.4e-05 ***** |

Supplementary Table 13: Gut microbiota: ANCOVA testing the influence of treatment and/or time on microbiota composition (alpha-diversity)

| dpr | 0 | 2 | 4 | 6 | 8 | 10 | 12 | 14 | 30 | 45 |
| --- | --- | --- | --- | --- | --- | --- | --- | --- | --- | --- |
| AB | 0.386 | 0.052 | **0.043** | **0.039** | 0.636 | **0.025** | 0.094 | 0.717 | **0.025** | 0.501 |
| SP | 0.204 | **0.013** | **0.035** | **0.006** | 0.302 | **0.017** | 0.299 | **0.015** | **0.009** | 0.308 |
| AB:SP | 0.826 | 0.943 | 0.261 | 0.267 | 0.602 | 0.883 | 0.084 | **0.039** | 0.251 | 0.411 |

Supplementary Table 14: Beta-diversity: ANOVA p-values evaluating the MANOVA model on the principal components (PCs) of the external whole-body microbiome of juveniles at each time point. Comparison of Antibiotics (AB) treatment groups (Antibiotics and AntiSpike) and Spike (SP) treatment groups (Spike and AntiSpike).

| dpr | 0 | 2 | 4 | 6 | 8 | 10 | 12 | 14 | 30 | 45 |
| --- | --- | --- | --- | --- | --- | --- | --- | --- | --- | --- |
| AB | 0.646 | 0.399 | 0.435 | 0.185 | 0.245 | 0.875 | 0.135 | 0.923 | 0.332 | 0.218 |
| SP | 0.325 | 0.467 | 0.488 | 0.941 | 0.591 | 0.291 | **0.044** | 0.924 | 0.366 | 0.558 |
| AB:SP | 0.471 | 0.290 | 0.244 | 0.336 | 0.422 | 0.975 | 0.481 | 0.348 | 0.609 | 0.707 |

Supplementary Table 15: Beta diversity: ANOVA p-values evaluating the MANOVA model on the principal components (PCs) of the internal sterile gut microbiome of juveniles at each time point. Comparison of Antibiotics (AB) treatment groups (Antibiotics and AntiSpike) and Spike (SP) treatment groups (Spike and AntiSpike).

**Experiment 3 (C) – Spike community**

|  | Placenta-like tissue | Brood pouch swabs | Male gut | Male testes | Ovipositor | Female gut |
| --- | --- | --- | --- | --- | --- | --- |
| All strains | **0.021** | **0.044** | 0.217 | 0.637 | 0.250 | 0.080 |
| *Kiloniella* | 0.433 | 0.321 | 0.532 | *n/a* | 0.368 | 0.392 |
| *Marinomonas* | *n/a* | 0.392 | *n/a* | *n/a* | *n/a* | *n/a* |
| *Sulfitobacter* | 0.091 | 0.760 | 0.817 | 0.542 | 0.286 | 0.082 |
| *Vibrio* | 0.258 | 0.240 | 0.219 | 0.392 | 0.392 | 0.708 |
| *Shewanella* | 0.392 | **0.018** | 0.392 | 0.392 | 0.179 | 0.430 |

Supplementary Table 16: Kruskal-Wallis H test p-values indicate impact of spike community strains on adult tissues of S. typhle. Data is based on normalized counts.
