# Supplementary material for: Paternal microbiota manipulation influences offspring microbial colonization and development in a sex role-reversed pipefish
Source: Sci Rep. 2025 Aug 22;15:30911. doi: 10.1038/s41598-025-16222-y (PMC12373867; doi:10.1038/s41598-025-16222-y)
Supplement: Supplementary file 2 — Supplementary Material 2 [file 41598_2025_16222_MOESM2_ESM.docx]

Paternal microbiota manipulation influences offspring microbial colonization and development in a sex role-reversed pipefish

Kim-Sara Wagner, Frédéric Salasc, Silke-Mareike Marten & Olivia Roth

**Supplementary Figures:**

**Experiment 1: Cultivation and characterization of sex-specific microbiota**


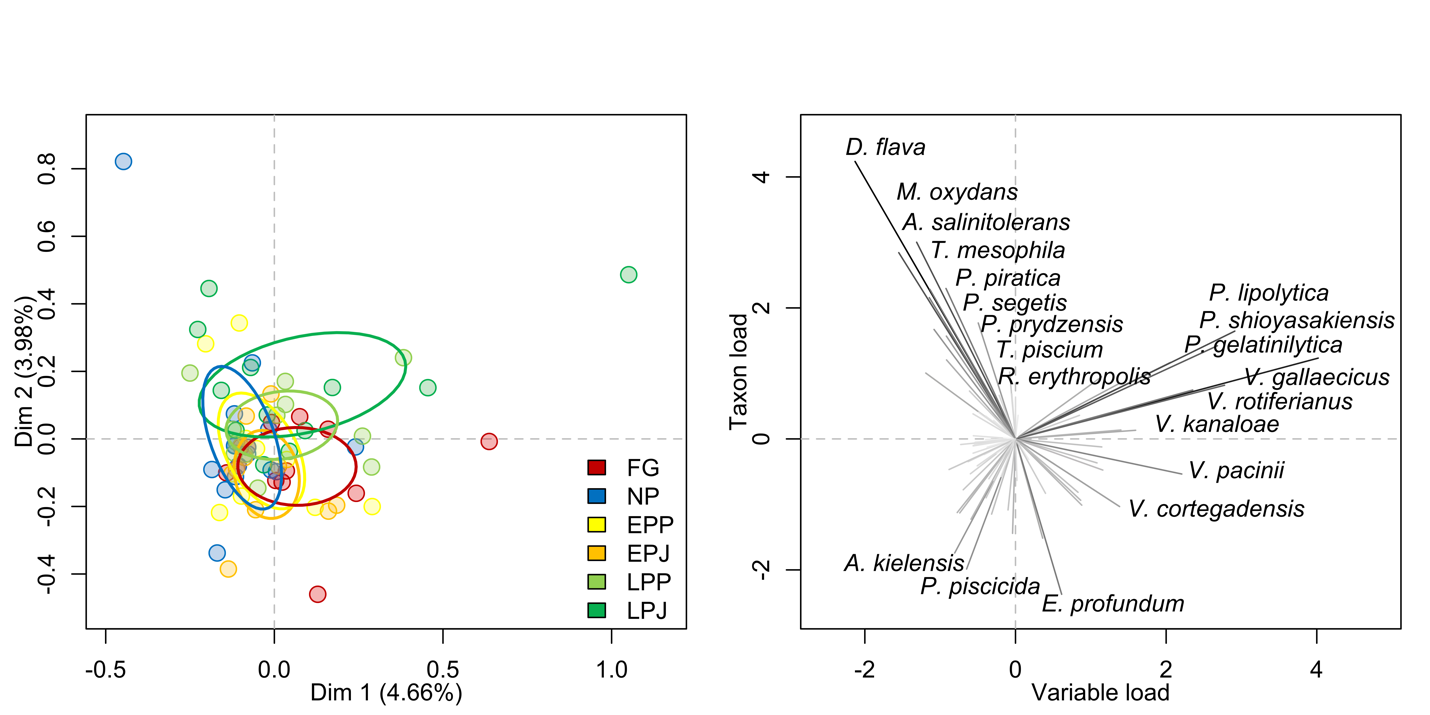


Supplementary Figure 1: Multiple correspondence analysis (MCA) of six groups for dimensions 1 and 2 (A) and factor map displaying the loadings retained by the isolated bacterial species (B). Ellipses include 40% of data of the respective group. Colors represent sex and pregnancy stages as described above.

**Experiment 2: Effectiveness of antibiotics for natural pipefish microbiota depletion**

Supplementary Figure 2: Well diffusion test to test for antibiotics in the water. Each well number corresponds to its respective tank. The center well was used for a control sample. Pictures were taken after 12 hours of incubation. Control (A) and Kanamycin (B) show no zones of inhibition, whereas treatments containing Chloramphenicol (C and D) show clear zones. Pictures show plates from beginning of the experiment (T0), after 24 hours (T24) and 48 hours (T48). For C and D additionally time points T6 and T12.

Supplementary Figure 3: Impact of antibiotics (mean + SE) on Shannon diversity of female ovipositor microbiota (A) and on relative abundance of spike community strains on the ovipositor tissue (B-F) over a period of 312 hours. Colours indicate treatment groups: Control (green), Kanamycin (yellow), Chloramphenicol (red) and Mix (purple). Red vertical line represents time of treatment dose (48 hours after treatment start). Samples were taken at the time points indicated on the x-axis; data points are horizontally shifted to display individual variation and avoid overlap.


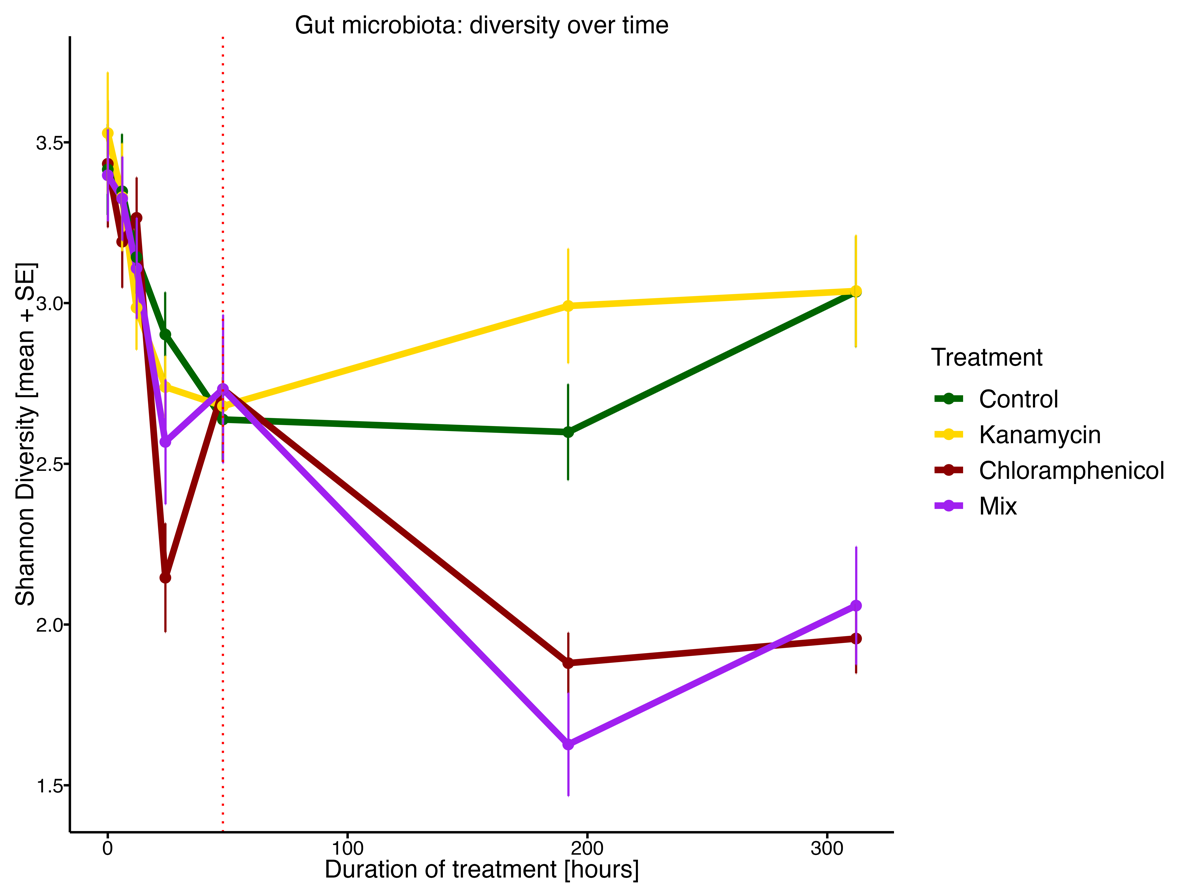


Supplementary Figure 4: Alpha diversity changes over time under the influence of different antibiotic treatments in the gut microbiota of S. typhle. Colors represent treatment groups and data points display mean diversity ± standard error.

Supplementary Figure 5: Pseudoalteromonas was initially selected as a paternal indicator species within the spike community but could not be depleted in either brood pouch nor ovipositor tissue by chloramphenicol and was therefore excluded for further experiments. Samples were taken at the time points indicated on the x-axis; data points are horizontally shifted to display individual variation and avoid overlap.


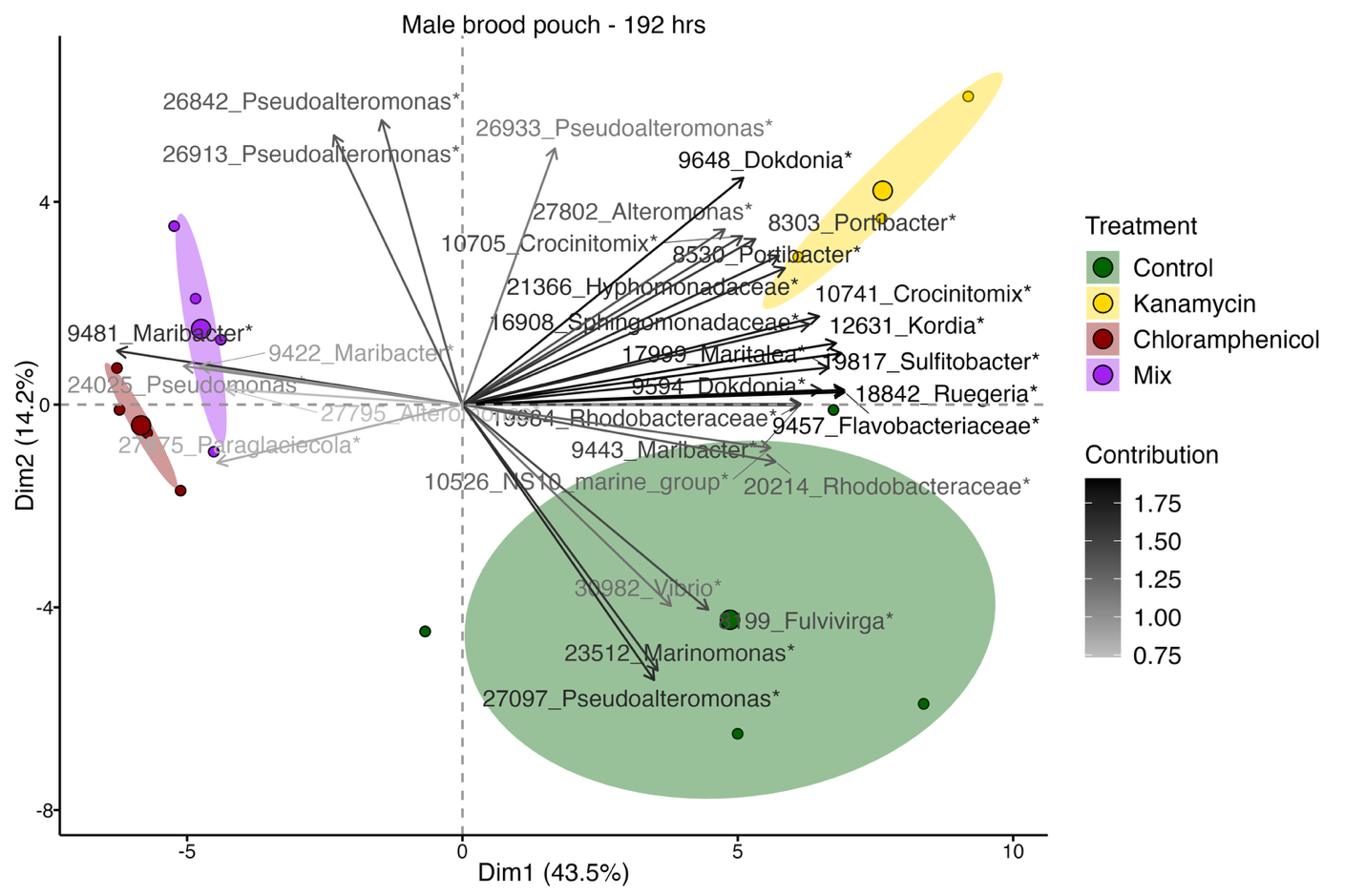


Supplementary Figure 6: PCA Biplot of the sex-specific microbiota under the influence of different antibiotics at 192 hours of treatment. Ellipses display a 95% confidence interval around the group’s mean. Colours represent treatment groups. Factor map displays the ASV ID and the genus name, respectively family name if genus could not be identified.


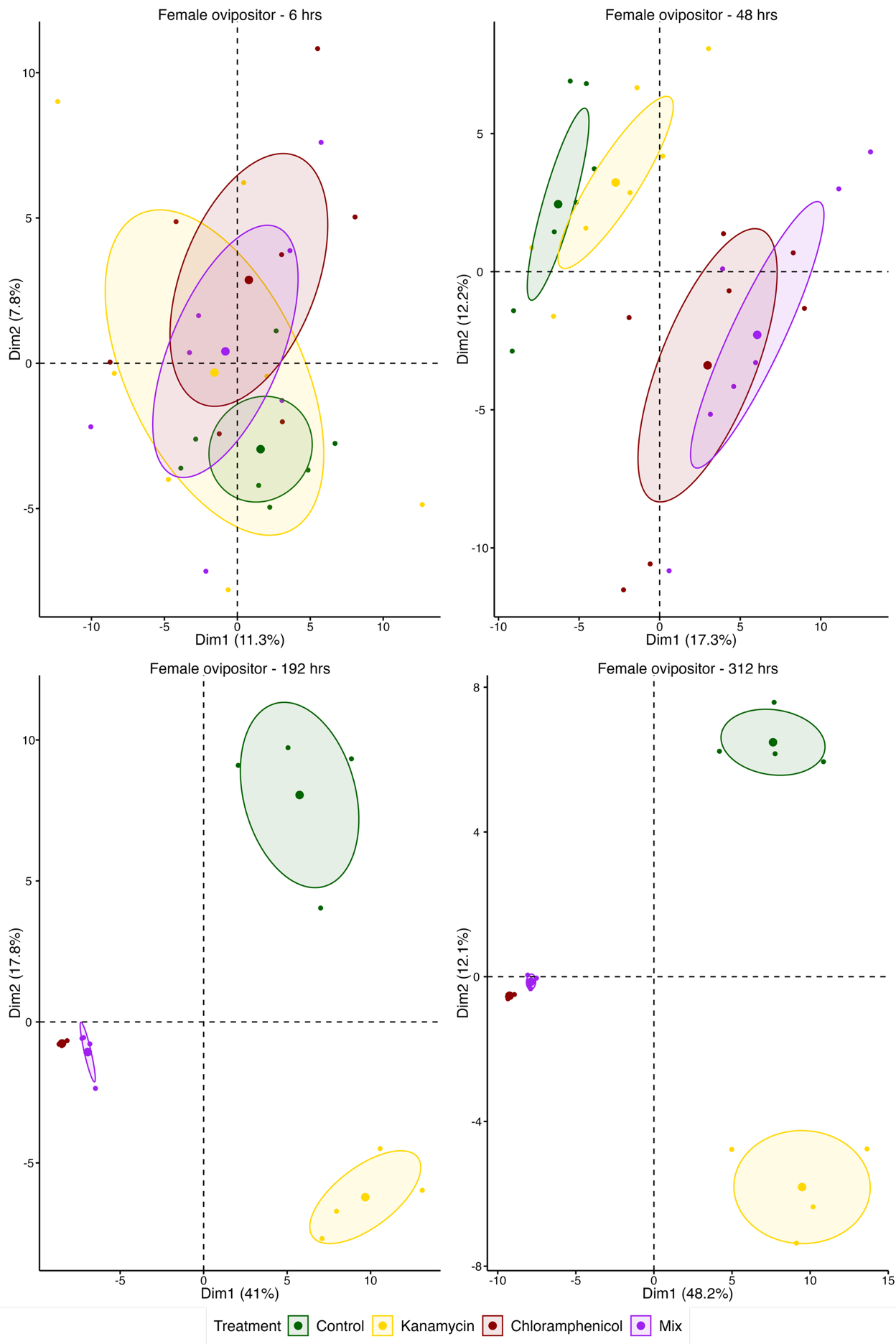


Supplementary Figure 7: Principal component analyses (PCAs) showing the effects of antibiotic treatment on the microbiota of the female ovipositor at different time points. Panels A and B (6 h and 48 h) represent the treatment phase, while panels C and D (192 h and 312 h) show the maintenance phase. PCAs are based on normalized, log-transformed ASV data (ASVs present in >20% of samples). Colors indicate treatment groups: Control (green), Kanamycin (yellow), Chloramphenicol (red), and Mix (purple). Ellipses represent 95% confidence intervals around the group centroid.

**Experiment 3: Manipulating paternal sex-specific microbiota to unravel impact on the offspring**

Supplementary Figure 8: Composition of spike community mixed culture that was used in 30x concentration for treating the male brood pouch. Bars represent sequencing counts of selected candidate strains within the culture.


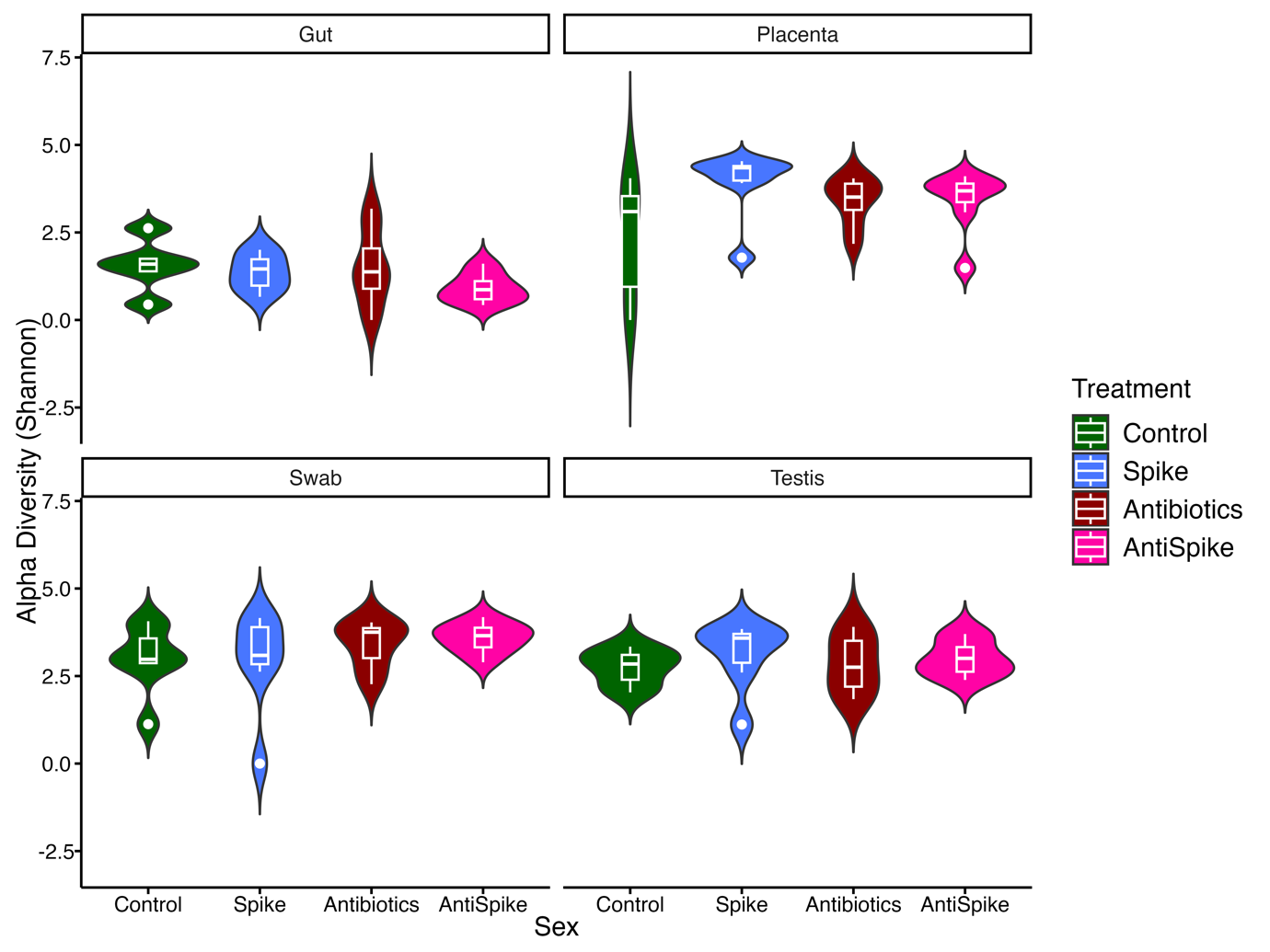


Supplementary Figure 9: Violin and boxplots showing alpha diversity of male organs under the influence of different treatments. Panels indicate from which organ the microbiota was sampled and sequenced.

Supplementary Figure 10: Female microbiome composition of ovipositor and gut, relative abundance of spike bacteria and counts of individual spike strains. PCA biplots displaying treatment effects (antibiotics and spike) on female tissue. Colours represent the paternal treatment group: green = Control, blue = Spike, red = Antibiotics, pink = AntiSpike, Ellipses represent 95% confidence intervals around the group centroid. Factor map shows ASV ID and genus name respectively family name. boxplots show the relative abundance (log scale) of spike bacteria within the whole microbiome of the respective tissue. Colours represent the paternal treatment group. Lower heatmaps display normalized individual sample counts; darker red indicates higher bacterial abundance. In the heatmaps, samples are grouped by treatment in the following order (left to right): Control, Spike, Antibiotics, AntiSpike, followed by environmental sources where applicable: Water (W), Spike Water (SPW), Mysids, and Antibiotic-treated Mysids (Anti_Mysids).


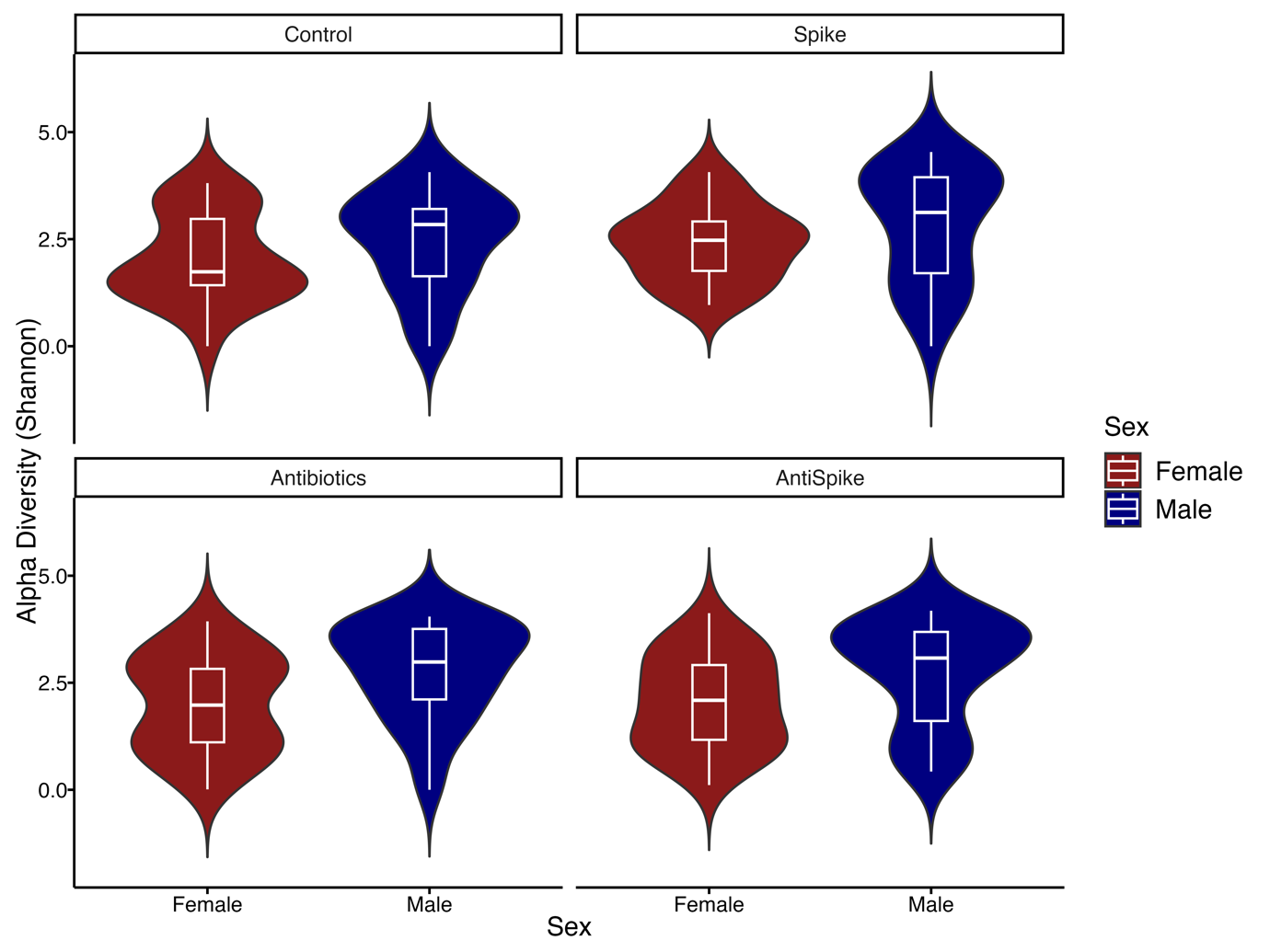


Supplementary Figure 11: Violin and boxplot showing an alpha diversity comparison of females (red) and males (blue) separated by treatment group.


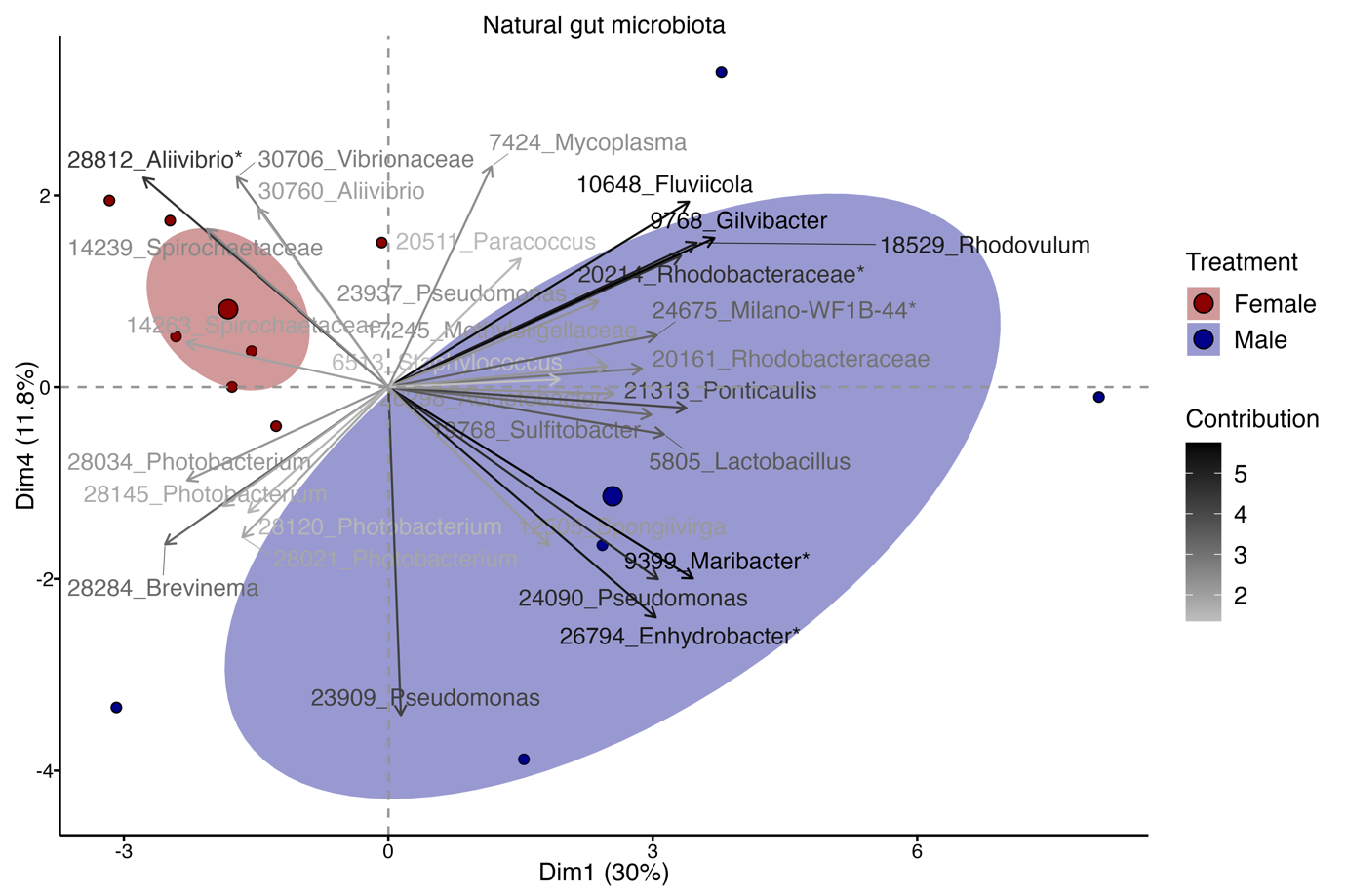


Supplementary Figure 12: The natural gut microbiota of male and female pipefish (Control group only) indicates a strong sexual dimorphism. Ellipses indicate a confidence interval around the group’s mean. Factor map shows ASV ID and genus.


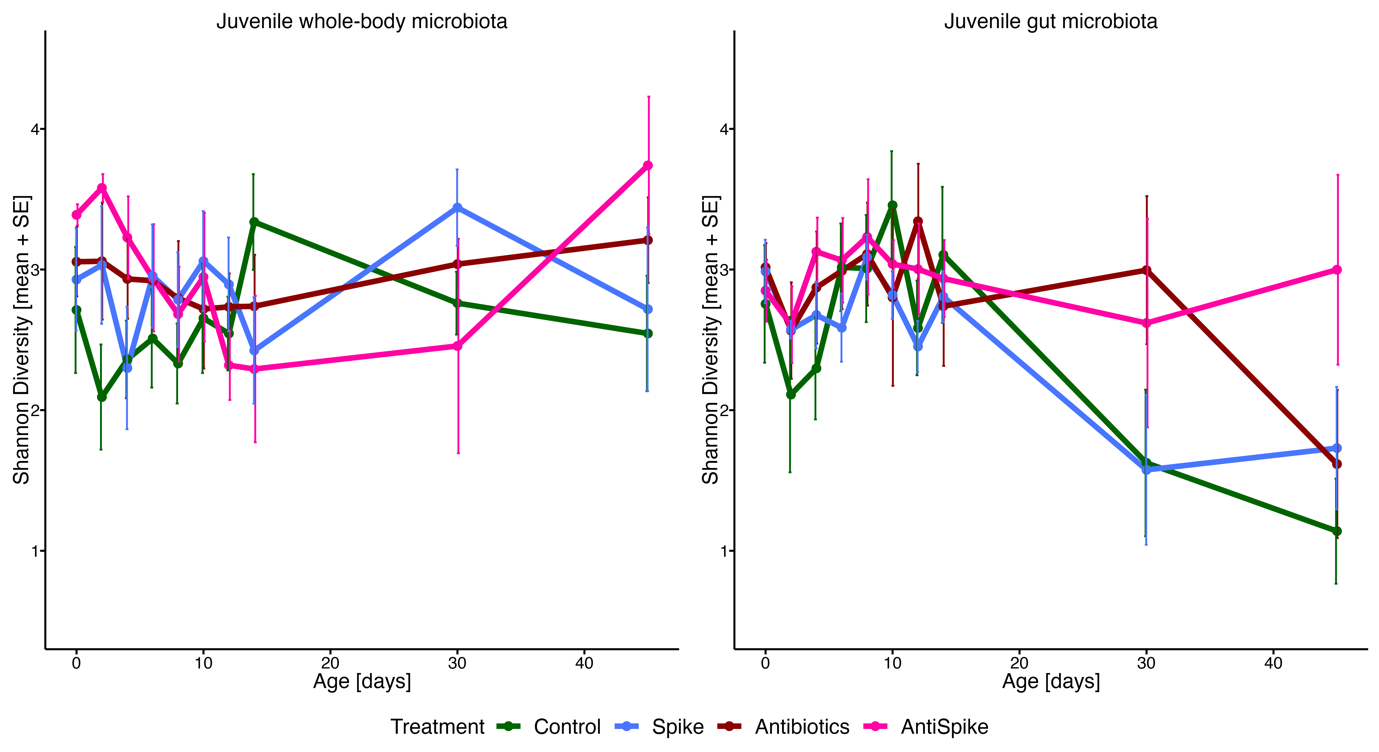


Supplementary Figure 13: Alpha diversity (Shannon index) of juvenile whole body (A) and gut (B) microbiota from birth until 45 days post release. Colors indicate paternal treatment groups. Data points represent mean diversity ± standard error (SE).

Supplementary Figure 14: Internal (gut) microbiota composition of juveniles from birth (A), 6 days (B), 10 days (C) up to 30 days (D) after birth. Colors represent paternal treatment. Ellipses show a 95% confidence interval around the group’s mean. Factor map shows ASV ID and genus name respectively family name if genus could not be identified.

Supplementary Figure 15: Relative abundance of spike community strains in the male gut (A) and the male testes (B). Heatmaps showing counts of individual strains for gut (C) and testes (D).

Supplementary Figure 16: Throughout the entire sampling period of juveniles, water (blue) and food (orange) samples cluster separately from the fish samples
